# Influence of the Microenvironment in the Transcriptome of *Leishmania infantum* Promastigotes: Sand Fly *versus* Culture

**DOI:** 10.1371/journal.pntd.0004693

**Published:** 2016-05-10

**Authors:** Pedro J. Alcolea, Ana Alonso, Mercedes Domínguez, Víctor Parro, Maribel Jiménez, Ricardo Molina, Vicente Larraga

**Affiliations:** 1 Laboratorio de Parasitología Molecular, Departamento de Microbiología Molecular y Biología de las Infecciones, Centro de Investigaciones Biológicas, Consejo Superior de Investigaciones Científicas, Madrid, Spain; 2 Unidad de Inmunología Microbiana, Centro Nacional de Microbiología, Virología e Inmunología Sanitarias, Instituto de Salud Carlos III, Majadahonda, Madrid, Spain; 3 Laboratorio de Ecología Molecular, Centro de Astrobiología, Instituto Nacional de Técnica Aeroespacial “Esteban Terradas”—Consejo Superior de Investigaciones Científicas, Torrejón de Ardoz, Madrid, Spain; 4 Unidad de Entomología Médica, Servicio de Parasitología, Centro Nacional de Microbiología, Virología e Inmunología Sanitarias, Instituto de Salud Carlos III, Majadahonda, Madrid, Spain; Lancaster University, UNITED KINGDOM

## Abstract

Zoonotic visceral leishmaniasis is a vector-borne disease caused by *Leishmania infantum* in the Mediterranean Basin, where domestic dogs and wild canids are the main reservoirs. The promastigote stage replicates and develops within the gut of blood-sucking phlebotomine sand flies. Mature promastigotes are injected in the dermis of the mammalian host and differentiate into the amastigote stage within parasitophorous vacuoles of phagocytic cells. The major vector of *L*. *infantum* in Spain is *Phlebotomus perniciosus*. Promastigotes are routinely axenized and cultured to mimic *in vitro* the conditions inside the insect gut, which allows for most molecular, cellular, immunological and therapeutical studies otherwise inviable. Culture passages are known to decrease infectivity, which is restored by passage through laboratory animals. The most appropriate source of promastigotes is the gut of the vector host but isolation of the parasite is technically challenging. In fact, this option is not viable unless small samples are sufficient for downstream applications like promastigote cultures and nucleic acid amplification. In this study, *in vitro* infectivity and differential gene expression have been studied in cultured promastigotes at the stationary phase and in promastigotes isolated from the stomodeal valve of the sand fly *P*. *perniciosus*. About 20 ng RNA per sample could be isolated. Each sample contained *L*. *infantum* promastigotes from 20 sand flies. RNA was successfully amplified and processed for shotgun genome microarray hybridization analysis. Most differentially regulated genes are involved in regulation of gene expression, intracellular signaling, amino acid metabolism and biosynthesis of surface molecules. Interestingly, meta-analysis by hierarchical clustering supports that up-regulation of 22.4% of the differentially regulated genes is specifically enhanced by the microenvironment (i.e. sand fly gut or culture). The correlation between cultured and naturally developed promastigotes is strong but not very high (Pearson coefficient R^2^ = 0.727). Therefore, the influence of promastigote culturing should be evaluated case-by-case in experimentation.

## Author Summary

The protozoan parasite *Leishmania infantum* causes visceral leishmaniasis in humans and is responsible for a recent outbreak reported in central Spain. Domestic dogs and wild canids are the main reservoirs. The life cycle of the parasite involves two stages and two hosts. The motile promastigote stage differentiates within the gut of the sand fly vector host and develops into non-motile amastigotes within phagocytes of the mammalian host. Promastigotes are routinely cultured in liquid media because it is assumed that they mimic the conditions within the gut of the insect. Therefore, the culture model is used in most studies about the biology of the parasite, pathogenesis and development of vaccines and new compounds for treatment. Isolating promastigotes from the natural microenvironment (i.e. the vector host) is desirable but technically challenging. We were able to perform a high-throughput analysis of gene expression thanks to mRNA amplification. The over-expressed genes detected may influence life cycle progression depending on the promastigote microenvironment (i.e. culture or vector host). Upcoming studies based on these results may reveal new therapeutic targets or vaccine candidates. Our results suggest that evaluating the influence of cultures in experimentation is convenient.

## Introduction

The genus *Leishmania* (Kinetoplastida: Trypanosomatidae) is responsible for leishmaniasis, a vector-borne parasitic disease with an estimated prevalence of 12 million people worldwide. Visceral leishmaniasis is the most severe form. It is fatal if left untreated. In fact, it causes about 60,000 deaths annually [1,2]. *L*. *infantum* is the etiological agent of zoonotic visceral leishmaniasis in the Mediterranean Basin, where co-infection with the HIV has been reported [3,4]. The main reservoirs of *L*. *infantum* are domestic dogs and wild canids. However, hares and rabbits have been involved as reservoirs in an outbreak in humans reported in the southwest of the Autonomous Community of Madrid [5,6,7].

The life cycle of the parasite is digenetic and involves an insect stage (promastigote) and a vertebrate stage (amastigote). Promastigotes replicate and differentiate inside the gut of hematophagous female sand fly vectors (Diptera: Psychodidae, Phlebotominae) that innoculate metacyclic promastigotes into the mammalian host´s dermis when feeding. Promastigotes engulfed by phagocytes are able to develop into amastigotes, which multiply inside parasitophorous vacuoles. The proven vectors of *L*. *infantum* in Spain are *Phlebotomus perniciosus* and *P*. *ariasi* [8]. The former is the major vector in the central and the western Mediterranean Basin [9].

In the 1960s and 70s, axenic culture of promastigotes was developed in undefined media. To some extent, this procedure allows obtaining biomass and reproducing *in vitro* the conditions inside the gut of the sand fly [10,11,12,13]. In fact, promastigote cultures are grown at 26–27°C (reviewed in [14,15]). The axenic culture model is more stable and reproducible in promastigotes than in amastigotes [16,17]. Conversely, obtaining promastigotes from the gut of the sand fly is technically challenging. Established laboratory colonies are required and the promastigote biomass isolated is usually insufficient for subsequent procedures. For these reasons, promastigotes are generally cultured. The original features, infectivity and virulence of the parasite become attenuated after numerous culture passages, which is often remedied by passages through laboratory animals (reviewed in [14]). Axenic culture does not influence genome content analysis and other types of studies. However, others are affected, such as parasite-host cell interaction and immunological studies.

RNA polymerase II transcription is performed without a canonical promoter for each gene in *Leishmania* spp. Protein coding genes are arranged in long polycistronic gene clusters (PGCs) [18,19,20] that are constitutively transcribed. The steady-state transcript levels are post-transcriptionally regulated [21]. Relatively low differential gene expression rates have been described in these organisms [22]. Differential gene expression profiling studies provided data about relative transcript abundance of hundreds of genes, as well as valuable information about the biology of *Leishmania* spp. (e.g., a succession of transient and permanent changes in gene expression during differentiation of promastigotes to amastigotes [23] and the relevance of temperature increase and acidification in this process [17]).

The purpose of this study is comparing *in vitro* infectivity and differential transcript abundance of promastigotes isolated from two different environments: the anterior thoracic midgut of the experimentally infected *P*. *perniciosus* sand flies (Pro-Pper) and stationary phase of axenic culture (Pro-Stat). Small amounts of RNA could be isolated and mRNA was amplified for subsequent microarray hybridization analysis. For obvious reasons, proteome analysis is not suitable so far [24]. The study contributes to explain the reliance of promastigote axenic cultures.

## Methods

### Ethics statement

Blood samples from a New Zealand White rabbit were required for infecting *P*. *perniciosus* with *L*. *infantum*. Breeding, handling and sampling was performed following the EU (2010/63) and Spain (RD1201/2005) regulations. The ISCIII Ethics Committee for Research in Animal Welfare approved the blood extraction protocol included in the CBA PA73-2011 license.

### Axenic culture of promastigotes

The *Leishmania infantum* isolate MCAN/ES/98/10445 (zymodeme MON-1) was axenically cultured. The inocula were aliquots of mid logarithmic phase promastigote cultures at the fourth passage after extraction from the gut of experimentally infected sand flies (see below). These aliquots were cryopreserved at -196°C in heat inactivated fetal bovine serum (HIFBS) containing 10% DMSO. Three independent biological replicate cultures were performed at 27°C in complete medium, which contained RPMI 1640 supplemented with L-glutamine (Cambrex, Karlskoga, Sweden), 10% HIFBS (Cambrex) and 100 μg/ml streptomycin– 100 IU/ml penicillin (Cambrex). After 72 h, promastigote samples were harvested at 2000 x g for 10 min and processed daily (see below). Only stationary phase promastigote samples obtained the day before the beginning of the death phase (Pro-Stat) were subsequently analyzed. *In vitro* infection of the human histiocytic leukemia U937 cell line [25] (ATCC CRL1593.2) and high-throughput differential gene expression analysis were performed with Pro-Pper and Pro-Stat.

### *In vitro* infection of phagocytes

The U937 cells were cultured at 37°C in complete medium in 175 cm^2^ flasks in an atmosphere of 5% CO_2_ for 72 h. Once the cells had been harvested at 250 x g for 10 min, differentiation was triggered with 20 ng/ml phorbol 12-myristate 13-acetate (Sigma, Saint Louis, MO) in complete medium for 72 h [26]. Then, the cells were mildly rinsed with RPMI 1640 supplemented with L-glutamine (Cambrex) and detached by vigorous shaking in the presence of 0.5 g/l trypsin, 0.2 g/l EDTA (Cambrex). Trypsin inactivation was carried out by adding one volume of complete medium. Next, phagocytes were centrifuged and mixed with stationary phase promastigotes at a proportion of 1:20. Infection was allowed at 37°C in complete medium in a water bath for 2 h. During incubation, the mixture was mildly shaken every 15 min. Then, the cells were centrifuged and incubated in complete medium at 37°C in an atmosphere of 5% CO_2_ for 72 h. The cultures were rinsed with complete medium after 2 and 16 h post-infection in order to eliminate remaining promastigotes. This procedure was used to obtain infected phagocytes for subsequent infection of sand flies (see below).

The *in vitro* infection assays were performed following a similar procedure but on 8-well cell chamber slides (LabTek, New York, NY). In this case, Pro-Pper and Pro-Stat were added directly onto the stimulated U937 cells attached to the surface (1:5). Therefore, detachment of phagocytes with trypsin was not required. Fixation and staining were performed 48 h post-infection. First, the wells were rinsed with fresh complete medium by thorough pipetting. Then, the cells were treated with hypotonic solution (complete medium:water 9:11) for 5 min. Next, four washes with 150 μl ethanol-acetic acid 3:1 were carried out. The cells were fixed with the same solution for 10 min three times. The preparations were allowed to air dry and the wells were removed from the slide. Modified Giemsa staining was performed with Diff-Quick Stain Solution I and II (Dade Behring, Marburg, Germany). The preparations were rinsed with distilled water, air dried and mounted with Entellan Neu (Merck, Darmstadt, Germany). Finally, the number of amastigotes (Amas) per infected cell was estimated by counting 100 cells per biological replicate randomly. The experiment was performed in triplicate. The differences in infectivity between Pro-Pper and Pro-Stat were statistically assessed by the paired Student's t-test.

### Infection of *Phlebotomus perniciosus* and isolation of Pro-Pper promastigotes from the anterior thoracic midgut

An established colony of *P*. *perniciosus* was maintained at 27–28°C, 90–100% relative humidity, 17 h light– 7 h darkness photoperiod and 30% fructose solution in a climatic chamber. Two million U937 cells from an infected culture (see above) were resuspended in 2 ml defibrinated rabbit blood. The mixture was used to feed 150–200 female sand flies over a 3-day chicken skin membrane [27]. Then, the sand flies were maintained at 27–28°C, 90–100% relative humidity, 17 h light—7 h darkness photoperiod in a climatic chamber, obviously in the absence of the fructose solution. The course of infection was followed every day. For this purpose, a sample of sand flies was dissected using entomological needles and the guts were removed and studied at the light microscope (40X). Once promastigotes reached the stomodeal valve in the monitoring samples, twenty sand flies per experimental replicate sample were dissected in a PBS drop. Then, the entire guts were isolated and the anterior thoracic midgut was separated in a new PBS drop on the same slide. Only this portion was slightly pressed with a coverslip, and the drop containing promastigotes (Pro-Pper) in suspension was recovered with a Pasteur pipette. Hence, carryover of gut tissue was minimized as much as possible. Finally, the sample was directly added onto stimulated U937 cells attached to the well in the case of the infection experiment, or centrifuged at 4°C and washed in PBS for subsequent RNA isolation (see below). After 72 h of development, the RNA samples were prepared daily until sand flies began to die as a consequence of infection. Therefore, Pro-Pper is defined as the promastigotes samples isolated from the sand fly anterior thoracic midgut behind the stomodeal valve one day before the beginning of the dead phase. This criterion was also applied for preparation of Pro-Stat, thus ensuring that both populations are comparable.

### RNA isolation, mRNA amplification and synthesis of labeled cDNA

Immediately after isolation of Pro-Pper and Pro-Stat, RNA was purified by extraction with 0.5 ml TRizol reagent (Life Technologies, Carlsbad, CA) following the manufacturer’s instructions. One μg/ml glycogen (Life Technologies) was added as carrier prior to RNA precipitation with isopropanol. At this point, RNA was stored at -80°C until use. Two mRNA amplification rounds were performed with MessageAmp II aRNA Amplification Kit (Life Technologies) as described [28]. RNA quality was assessed by conventional agarose gel electrophoresis and Experion RNA HighSens Analysis Kit (Bio-Rad Laboratories, Hercules, CA) according to the manufacturer's instructions. In the first case, the electrophoresis cell, tray and comb were rinsed with hydrogen peroxide and the aaRNA samples were run at 5 V/cm in a 1.5% agarose gel prepared with RNase-free water. The gel was pre-stained with GelRed Nucleic Acid Gel Stain (Biotium, Hayward, CA) diluted 1:10,000.

The first strand aminoallyl-cDNA was synthesized. First, 10 μg of aaRNA were mixed with 6 μg of random hexamer primers (Life Technologies). The mixture was denatured at 70°C for 10 min and immediately cooled on ice. Thereafter, samples were incubated at 46°C for 3 h with 230 μM dTTP, 340 μM aminoallyl-dUTP, 570 μM (each) dATP, dCTP and dGTP, 10 μM DTT and 600 U SuperScript Reverse Transcriptase (Life Technologies) in a final reaction volume of 30 μl. Next, RNA was degraded at 70°C for 30 min in 100 mM NaOH/10 mM EDTA. The solution was neutralized with 3 μl of 3 M sodium acetate pH 5.2. Then, cDNA was purified with QiaQuick PCR Purification Kit (Qiagen, Hilden, Germany). The manufacturer’s instructions were followed except for using phosphate wash buffer (5 mM KPO_4_, 80% ethanol, pH 8.0) and phosphate elution buffer (4 mM KPO_4_) instead of the respective commercial buffers. Then, the purified aminoallyl-cDNA samples were completely dried in a vacuum centrifuge and resuspended in 10 μl of water. Five μl of Cy3 or Cy5 (respectively for Pro-Stat and Pro-Pper) monofunctional dye (GE Healthcare, Chalfont Saint Giles, UK) dissolved in DMSO at 12 ng/μl were added. Coupling was allowed at room temperature in darkness for 1 h. Finally, the labeled cDNA samples were purified with QiaQuick PCR Purification Kit (Qiagen) according to the manufacturer's instructions.

### Microarray hybridization and data analysis

Whole genome shotgun DNA microarrays of *L*. *infantum* (GEO Accession number GPL6781) were soaked with 0.1% N-lauroylsarcosine in 2XSSC; then in 2XSSC. After denaturation at 95°C for 3 min, they were fixed in chilled 100% ethanol and spin-dried in a slide mini centrifuge. A 60 μl drop containing 2XSSC, 0.3% N-lauroylsarcosine, 60 mM Tris-HCl pH8.0, 83 ng/ml denatured herring sperm DNA and 1% BSA was deposited over a Hybri-Slip coverslip (Sigma). The slide was attached on the coverslip. Blocking was allowed at 42°C in a hybridization chamber submerged in a water bath for 30 min. Thereafter, labeled cDNA samples were mixed in equimolar amounts of each dye (50 pmol) and incubated at 40°C with blocked microarrays for 16 h (same as blocking solution except for 0.1% BSA, 25 ng/ml poly(T), 50% deionized formamide). Finally, the slides were soaked with 2XSSC, 0.2% SDS at 40°C and consecutively in 1XSSC and 0.2XSSC at room temperature.

Genomic DNA was isolated from non-infected sand flies by phenolic extraction as described [29] and directly labeled with Cy5 using GenomiPhi DNA Amplification Kit (GE Healthcare). For this purpose, 350 μM each dATP, dCTP, dGTP and [1/3 Cy5-dUTP, 2/3 dTTP] mix were used. Next, it was hybridized with the microarrays as a cross-hybridization control.

Hybridization data were acquired with a GenePix 4100A scanner (Axon, Foster City, CA). Raw data of local feature background medians were subtracted with GenePix Pro 7.0 software. The LOWESS per pin algorithm was applied for data normalization and differential expression was contrasted by the Student’s t-test. The AlmaZen software (BioAlma, Tres Cantos, Spain) was used for both purposes. The cutoff values for differential gene expression were the following: (i) fold change F ≥ 2 (Cy5/Cy3 ratio if Cy5 > Cy3) or F ≤ -2 (-Cy3/Cy5 ratio if Cy3 > Cy5), (ii) total relative fluorescence intensity value > 5000 arbitrary fluorescence units and (iii) p < 0.05. Three biological replicates were considered in the experiment.

### Identification of differentially regulated genes

The clones that complied with the cutoff values mentioned above were sequenced with the M13-pUC18 primers and assembled as described [29]. Correctly assembled clones fulfilled the following: (i) e-value < 1e-10 for both ends, (ii) convergent orientation in the genome sequence and (iii) length ≤ 11 kbp, according to the features of the genome library used for microarray construction [29]. The analyzed clones were classified in three categories: *a* clones (only a pair of alignments complies with all three conditions), *b* clones (more than a pair does due to adjoining sequence repeats; only the best sequence identity is considered) and *c* clones (not complete fulfilment of the requirements mainly due to the presence of two or more inserts in the clone). Then, clones were associated to genes annotated in the genome by using a Perl script that excludes 5% of the ORF end sequence that overlaps with the boundaries of the clone. Clones that do not fulfill this criterion but align with less than 5% of the length of a given annotated ORF were identified using the genome browser [29]. Clones that do not map with any ORF were aligned with complete transcript sequences including UTRs that were obtained by RNAseq in *L*. *major* [30]. Genes were classified in functional categories according to the Gene Ontology database (GO) and associated to EC identifiers and KEGG pathways [31] with BLAST2GO software [32]. In addition, the GeneDB [33] and TriTrypDB [34] databases were useful to retrieve information about gene functions, as well as literature. CLUSTALW2 alignments allowed distinguishing between gene copies.

### Validation by real time quantitative RT-PCR (qRT-PCR)

Unlabeled single stranded cDNA was synthesized following the same procedure as for microarray hybridization but using a mixture stock of 10 mM each dNTP. Custom TaqMan MGB Assay-by-Design (specifically primers and FAM-NFQ MGB probes, Life Technologies) were mixed with 1:5 serial dilutions of cDNA samples (10, 2 and 0.4 ng cDNA per reaction) and with TaqMan Universal Master Mix 2X (Life Technologies) in a final reaction volume of 10 μl. Primer and probe sequences are listed in S1 Table. The qRT-PCR reactions were run in a 7900HT Fast Real Time PCR system using the SDS 4.1. software (Life Technologies) following the procedure specified by the manufacturer. The thermal cycling conditions were: 95°C for 5 min; 40 x [95°C for 30”; 60°C for 1 min, data acquisition]. After checking coefficients of variation, PCR efficiencies were calculated by the standard curve best fit method using the data obtained in the triplicate dilution series experiment for each gene and cDNA sample (Pro-Pper/Pro-Stat). The normalized quantities were calculated by dividing the efficiency-corrected raw quantities (efficiency to the power of–Ct) for the gene of interest by those for the reference gene (*L*. *infantum* gGAPDH). Fold changes were obtained by dividing the normalized quantities (Pro-Pper/Pro-Stat). This procedure is based on specifications provided by Bookout et al. [35].

### Analysis of gene expression by clustering

The relative expression profiles of Pro-Pper/Pro-Stat were compared with the Pro-Pper/Amas [24], Pro-Stat/Pro-Log and Pro-Stat/Amas [28] ones. Pro-Log are defined as cultured promastigotes at early logarithmic phase (about 48 h in culture, hence undifferentiated). For this purpose, the TIGR's Experiment Viewer 4.9 (MEV) software was used. The normalized fold-change values of each microarray hybridization experiment were loaded. The hierarchical clustering-support tree (HCL-ST) algorithm was run using Euclidean metrics and setting jackknifing resampling, 100 iterations, the complete linkage method and the Pearson correlation coefficient as the distance metrics for the ST.

## Results and Discussion

### Isolation of Pro-Pper and evaluation of *in vitro* infectivity

**Fig 1:**
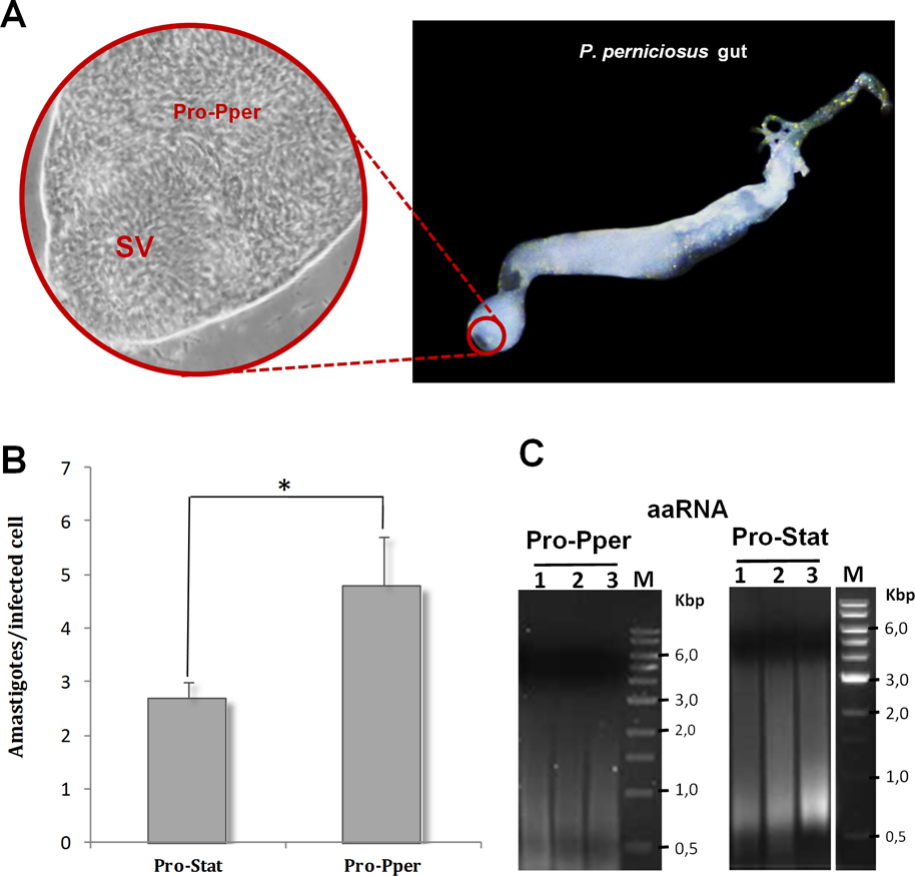
Sand fly gut dissection, *in vitro* infectivity and mRNA amplification. (A) Detail of Pro-Pper (40X) within the anterior thoracic midgut (10X). SV: stomodeal valve. Sand flies of an established colony were dissected to extract the whole guts. After that, the anterior part of the thoracic midgut, behind the SV, was separated in a PBS drop and slightly squeezed with a coverslip. Then, the PBS drop containing Pro-Pper in suspension was recovered, thus avoiding carryover of gut tissue as much as possible. (B) The U937 cell line was differentiated with phorbol esters on 8-well chamber slides and *in vitro* infected with *L*. *infantum* Pro-Pper and Pro-Stat (approximately 5 x 10^4^ promastigotes at a phagocyte:promastigote ratio 1:5 were added). The preparations were fixed and stained with modified Giemsa and 100 cells were randomly counted per replicate. The average number of amastigotes per infected cell was measured at 48 h post-infection. Mean ± SD: 2.7 ± 0.4 in the case of Pro-Stat and 4.8 ± 0.9 in the case of Pro-Pper. (C) Agarose gel electrophoresis of aaRNA samples used for the microarray analysis after synthesis of labeled cDNA. Total RNA was purified from isolated Pro-Pper immediately after dissection and doubly amplified (aaRNA) with MessageAmpII aRNA Amplification Kit (Life Technologies), due to sample amount requirements. Pro-Stat RNA was isolated and processed following the same procedure as for Pro-Pper. Five μl aliquots of the aaRNA samples were run at 5 V/cm in a 1.5% agarose gel prepared with RNase-free water after treatment of the electrophoresis cell, tray and comb with hydrogen peroxide. Three biological replicates of the microarray hybridization experiment were performed.

Infected sand flies were dissected to extract their digestive tracts. Next, the anterior part of the thoracic midgut, containing the stomodeal valve and Pro-Pper promastigotes, was isolated (Fig 1A). After slightly pressing with a coverslip, the PBS drop containing Pro-Pper promastigotes in suspension was recovered with a Pasteur pipette. Following this procedure, carryover of gut tissue was minimized as much as possible. Each Pro-Pper sample contained material from 20 sand flies. Three samples per condition were prepared for the *in vitro* infection and gene expression profiling experiments.

Pro-Pper has been defined above as the promastigote samples obtained from the sand fly anterior thoracic midgut behind the stomodeal valve the day before the dead phase began (day 6 in this case), whereas Pro-Stat are equivalent populations in stationary phase of axenic culture (day 7 in this case). Despite both are intrinsically heterogeneous, they are enriched in metacyclic promastigotes, especially Pro-Pper. The *in vitro* infection experiment of U937 cells has confirmed that Pro-Pper are significantly more infective than Pro-Stat (Student's t-test, p < 0.0001), as the mean ± SD of the number of amastigotes per infected cell at 48 h post-infection is 4.8 ± 0.9 and 2.7 ± 0.4, respectively (Fig 1B).

### mRNA amplification and microarray hybridization analysis of relative gene expression between Pro-Pper and Pro-Stat

**Fig 2:**
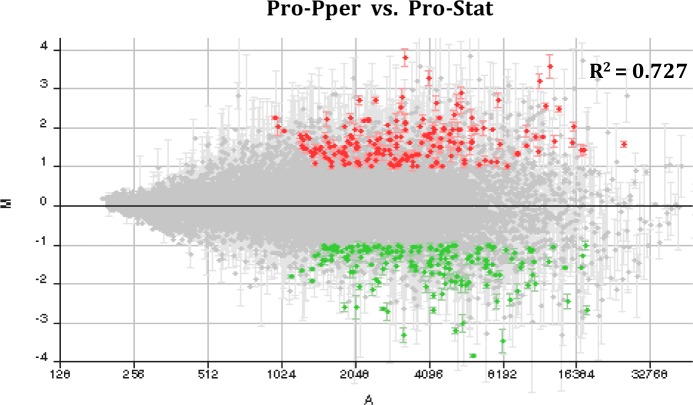
M/A scatter plot of the Pro-Pper/Pro-Stat microarray hybridization experiment. M = (log_2_Ri–log_2_Gi) and A = [(log_2_Ri + log_2_Gi)/2], where R and G are, respectively, red (Cy5) and green (Cy3) fluorescence intensity values previously normalized by the LOWESS per pin algorithm. Red spots correspond to selected clones containing at least a 2-fold up-regulated gene and green spots a 2-fold down-regulated one. Only statistically significant differences were selected (Student’s t-test, p<0.05). A fluorescence intensity filter was applied to select these data as well. The dot cloud is not dispersed and it is symmetric about the M = 0 line (lack of differential expression), as expected in a microarray analysis. The Pearson correlation coefficient between normalized fluorescence intensity values of Pro-Pper and Pro-Stat is R^2^ = 0.727. Consequently, both samples are strongly correlated but important differences are also observed.

All samples were immediately washed with PBS once and lysed with TRIzol reagent. The yield of total RNA isolation was about 20 ng per biological replicate sample in the case of Pro-Pper. For this reason, two rounds of mRNA amplification were performed to obtain enough starting material for microarray analysis. The Pro-Stat samples were prepared following the same procedure. The results of mRNA double amplification are shown in Fig 1C. Microarray hybridization data of control spots are included in S2 Table. As expected, the amastigote-specific A2 gene, spotted in the microarrays as a control gene [29], is not differentially expressed between Pro-Pper and Pro-Stat. The number of differentially regulated genes between Pro-Pper and Pro-Stat is 286: 148 are up-regulated in Pro-Pper and 138 in Pro-Stat (Fig 2, Table 1 and S3 Table). All clones that represent differentially regulated genes of known function are provided in S4 and S5 Tables, whereas Tables 2 and 3 contain only those discussed below. A complete explanation of Tables S4 and S5 is provided in S1 Text.

**Table 1:** Statistics of Pro-Pper/Pro-Stat differential gene expression profiles. Absolute frequencies of genes encoding proteins of known function and hypothetical proteins are provided, as well as type c clones and clones that overlap with more than one gene but that were not determined by qRT-PCR.

Annotation status	Frequency of differentially regulated genes in Pro-Pper/Pro-Stat	
	Up-regulated	Down-regulated
Genes of known function	79	57
Hypothetical protein genes	63	74
Type c clones/qRT-PCR N.D.	6	7
Total (n = 286)	148	138

**Table 2:** A selection of up-regulated genes of known function in Pro-Pper/Pro-Stat. Only the up-regulated genes of known function discussed in the subsection "Differential gene expression between Pro-Pper and Pro-Stat" are included in this table. The following items are specified for each selected clone: fold change (F ≥ 2); SD; Student’s t-test p-value; expect value in alignments (e-value); clone definition according to mapping outcomes a, b and c; Gene Id. retrieved from the database TriTrypDB [34]; functions annotated in the *L*. *infantum* genome sequence; qRT-PCR outcomes. See detailed information in the Methods section. S4 Table contains the complete set of up-regulated genes of known function.

*Clone*	*F*	*log*_*2*_*F ± SD*	*p*	*e-value*		*Def*.	*Id*. *(TriTrypDB)*	*Annotated gene function*	*qRT-PCR*	
				*Fw*	*Rv*					
Lin9E5	4.24	2.1 ± 0.1	0.001	0	0	b	LinJ.35.1150	Oligosaccharyl transferase-like protein		N.D.
Lin13C3	5.77	2.5 ± 1.0	0.049	3e-175	0	b	LinJ.21.0770	ATP-binding cassette subfamily E, member 1, putative (ABCE1)		N.D.
Lin13G4	3.12	1.6 ± 0.1	0.002	0	0	c	LinJ.29.0990	Signal peptidase, aspartic peptidase, Clan AD, family A22B, putative	+	2.5 ± 0.1
Lin22C9	3.75	1.9 ± 0.4	0.018	0	0	b	LinJ.33.2910	Ubiquitin-conjugating enzyme, putative (UBC)		N.D.
Lin31D4	3.22	1.7 ± 0.2	0.004	5e-131	0	b	LinJ.36.4230	Zinc-carboxypeptidase, putative, Clan MC, family M14		N.D.
Lin34F1	3.20	1.7 ± 0.4	0.017	0	0	b	LinJ.08.1000	Histone deacetilase, putative		N.D.
Lin49B6*	4.72	2.2 ± 0.3	0.008	0	0	b	LinJ.06.1320	Pteridine transporter, putative		N.D.
Lin72A2	2.16	1.1 ± 0.2	0.009	0	0	b	LinJ.36.0640	Sec14, cytosolic factor		N.D.
Lin76A1	6.02	2.6 ± 0.8	0.028	0	0	b	LinJ.31.3320	Histone H4, putative		N.D.
Lin76F1	7.81	3.0 ± 1.1	0.044	0	0	b	LinJ.34.3370	Phosphatidylinositol 4-kinase, putative		N.D.
Lin77B12	2.03	1.0 ± 0.1	0.002	0	0	b	LinJ.27.1520	Eukaryotic translation initiation factor eIF4E, putative		N.D.
Lin80B3	2.79	1.5 ± 0.2	0.005	0	0	b	LinJ.28.3250	Glucosamine-6-phosphate N-acetyltransferase, putative		N.D.
Lin80C3	2.00	1.0 ± 0.2	0.011	0	0	b	LinJ.28.3250	Glucosamine-6-phosphate N-acetyltransferase, putative		N.D.
Lin83C12	2.76	1.5 ± 0.4	0.020	0	0	b	LinJ.36.2160	Dolichyl-P-Man:GDP-Man5GlcNAc2-PP-dolichyl α-1,3-mannosyltransferase, putative (ALG3)		N.D.
Lin100E10	2.17	1.1 ± 0.2	0.013	2e-40	0	b	LinJ.35.3900	T-complex protein 1, η subunit, putative		N.D.
Lin100F4	2.48	1.3 ± 0.5	0.049	0	0	a	LinJ.28.2280	Dynein light chain LC6, flagellar outer arm, putative	+	16.8 ± 0.9
							LinJ.28.2290	A/G-specific adenine glycosylase, putative	+	2.0 ± 0.0
Lin100F12*	2.48	1.3 ± 0.5	0.049	0	0	a	LinJ.30.3040	Lsm5p, putative	+	3.4 ± 0.2
Lin102G11	2.60	1.4 ± 0.5	0.045	0	0	b	LinJ.13.1020	DNA-directed RNA-polymerase I subunit (RBP10), putative	+	14.2 ± 0.7
Lin103F1*	2.48	1.3 ± 0.2	0.008	0	0	a	LinJ.35.3080	Prenyl protein-specific carboxymethyltransferase, putative (ICMT)		N.D.
Lin106C12*	2.42	1.3 ± 0.3	0.023	0	0	b	LinJ.08.0010	Adaptor complex protein (AP) 3δ subunit 1, putative	+	9.1 ± 0.6
							LinJ.08.0030	Vesicle-associated membrane protein, putative	+	7.1 ± 0.3
Lin107C2	2.29	1.2 ± 0.1	0.004	0	0	b	LinJ.08.1000	Histone deacetilase, putative		N.D.
Lin111D8	4.80	2.3 ± 0.3	0.006	0	0	a	LinJ.08.1000	Histone deacetilase, putative		N.D.
Lin112A4	2.49	1.3 ± 0.4	0.030	1e-69	5e-72	b	LinJ.32.0050	Protein transport protein sec13, putative		N.D.
Lin112H3	3.79	1.9 ± 0.6	0.029	0	0	a	LinJ.29.2070	Protein farnesyltransferase α subunit, putative		N.D.
Lin113B9*	2.20	1.1 ± 0.1	0.006	0	0	b	LinJ.36.0570	U2 small nuclear ribonucleoprotein 16.5K, putative	+	10.4 ± 1.0
Lin123G3	9.84	3.3 ± 0.4	0.006	0	0	a	LinJ.23.0060	Cyclophilin, putative		N.D.
							LinJ.36.6780	Ubiquitin fusion degradation protein, putative (ubq)	+	2.3 ± 0.2
Lin134A9	4.93	2.3 ± 0.3	0.007	0	0	a	LinJ.36.2040	Nucleoside transporter 1, putative	+	2.4 ± 0.2
							LinJ.36.2050	Mismatch repair protein MSH6, putative	+	3.3 ± 0.1
Lin134E11	3.90	2.0 ± 0.4	0.011	0	0	a	LinJ.23.0060	Cyclophilin, putative		N.D.
Lin132E1	2.20	1.1 ± 0.1	0.005	0	0	c	LinJ.16.0450	Fucose kinase, putative	+	4.7 ± 0.4
Lin139D8	4.68	2.2 ± 0.4	0.009	0	0	b	LinJ.08.0010	Adaptor complex protein (AP) 3 δ subunit 1, putative		N.D.
Lin154G9	4.65	2.2 ± 0.3	0.007	0	0	b	LinJ.25.0080	Poly(A)-binding protein 3, putative (PABP3)		N.D.
Lin154H12	4.70	2.2 ± 0.2	0.003	0	0	b	LinJ.33.1770	UDP-GlcNAc:PI a1-6 GlcNAc-transferase		N.D.
Lin166F2	9.47	3.2 ± 0.8	0.021	1e-177	0	b	LinJ.21.0770	ATP-binding cassette subfamily E, member 1, putative (ABCE1)		N.D.
Lin168C4	3.78	1.9 ± 0.4	0.017	0	0	a	LinJ.36.3180	Clathrin coat assembly protein-like protein	+	2.0 ± 0.1
							LinJ.36.3190	pre-mRNA branch site protein p14, putative	+	43.2 ±1.5
Lin193F5*	4.31	2.1 ± 0.2	0.004	0	0	b	LinJ.36.2090	Ser/Thr protein phosphatase 2B, catalytic subunit A2, putative		
Lin208H5	7.31	2.9 ± 0.5	0.012	0	0	b	LinJ.28.2200	DNA-directed RNA polymerase-like protein, putative		N.D.
Lin269A6*	3.67	1.9 ± 0.4	0.012	0	0	a	LinJ.23.0060	Cyclophilin, putative		N.D.
Lin274G12	4.28	2.1 ± 0.2	0.003	0	0	a	LinJ.23.0040	β -propeller protein, putative	+	5.0 ± 0.2
							LinJ.23.0050	Peroxidoxin (Tryparedoxin peroxidase)	**-**	1.3 ± 0.1
Lin285F5	5.26	2.4 ± 0.9	0.041	0	0	a	LinJ.36.3610	Glycosyl transferase-like protein	+	7.2 ± 0.7

*The clone aligns with more gene sequences.

**Table 3:** A selection of down-regulated genes of known function in Pro-Pper/Pro-Stat. Only the down-regulated genes of known function discussed in the subsection "Differential gene expression between Pro-Pper and Pro-Stat" are included in this table. The following items are specified for each selected clone: fold change (F ≤ -2); SD; Student’s t-test p-value; expect value in alignments (e-value); clone definition according to mapping outcomes a, b and c; Gene Id. retrieved from the database TriTrypDB [34]; functions annotated in the *L*. *infantum* genome sequence; qRT-PCR outcomes. See more detailed information in the Methods section. S5 Table contains the complete set of down-regulated genes of known function.

*Clone*	*F*	*-log*_*2*_*[F]± SD*	*p*	*e-value*		*Def*.	*Id*. *(TriTrypDB)*	*Annotated gene function*	*qRT-PCR*	
				*Fw*	*Rv*					
Lin18A12	-2.32	-1.2 ± 0.5	0.046	0	0	b	LinJ.33.2430	UDP-glucose 4’-epimerase, putative		N.D.
Lin25B7	-2.73	-1.4 ± 0.2	0.008	0	0	b	LinJ.31.3390	Sodium stibogluconate resistance protein, putative		N.D.
Lin27C2	-2.78	-1.5 ± 0.5	0.040	0	0	b	LinJ.15.1070	Glutamate dehydrogenase, putative (GDH)		N.D.
Lin34B5	-2.06	-1.0 ± 0.1	0.004	2e-142	0	b	LinJ.03.0190	δ-1-pyrroline-5-carboxylate dehydrogenase, putative		N.D.
Lin34G5	-5.32	-2.4 ± 0.9	0.043	0	0	b	LinJ.06.0350	NAD(P)-dependent steroid dehydrogenase-like protein (NSDHL)	+	-2.0 ± 0.2
Lin35H4*	-3.57	-1.8 ± 0.4	0.013	0	0	b	LinJ.34.3740	Expression site-associated glycoprotein 5 (ESAG5)		N.D.
Lin50G2	-2.04	-1.0 ± 0.1	0.002	0	0	b	LinJ.34.2660	Amastin-like surface glycoprotein		N.D.
Lin55D10	-2.50	-1.3 ± 0.1	0.004	0	0	b	LinJ.18.1660	γ-glutamylcysteine synthetase, putative (GSH1)		N.D.
Lin57H5	-2.40	-1.3 ± 0.2	0.009	0	0	b	LinJ.08.0700	Amastin-like protein		N.D.
Lin80A1	-2.43	-1.3 ± 0.2	0.011	0	2e-65	b	LinJ.36.6550	Glucose transporter 2, putative		N.D.
Lin83D11	-2.22	-1.1 ± 0.4	0.046	0	0	b	LinJ.08.0700/10	Amastin-like protein		N.D.
Lin84B5	-2.04	-1.0 ± 0.3	0.032	0	0	b	LinJ.34.3030	α-keto acid decarboxylase, putative		N.D.
Lin91B12	-2.70	-1.4 ± 0.2	0.009	0	0	b	LinJ.34.2660	Amastin-like surface protein,putative		N.D.
Lin93C5	-4.32	-2.1 ± 0.4	0.009	0	1e-115	b	LinJ.13.1420	Pyrroline-5-carboxylate reductase (P5CR)		N.D.
Lin102E4*	-2.11	-1.1 ± 0.4	0.038	1e-156	0	b	LinJ.23.1200	Hydrophilic acylated surface protein A (HASPA1)		N.D.
Lin104F8	-2.71	-1.4 ± 0.3	0.011	0	3e-129	b	LinJ.24.1280	Amastin-like surface protein-like protein		N.D.
Lin110A4	-2.15	-1.1 ± 0.3	0.028	0	0	a	LinJ.24.1460	Mismatch repair protein, putative		N.D.
Lin123E6	-3.38	-1.8 ± 0.4	0.021	0	0	b	LinJ.23.0980	Actin-interacting protein		N.D.
Lin123D6	-2.72	-1.4 ± 0.1	0.001	0	0	b	LinJ.34.2660	Amastin-like surface protein, putative		N.D.
Lin125B1	-2.56	-1.4 ± 0.4	0.027	0	0	b	LinJ.23.1060	β-fructosidase/invertase/sucrose hydrolase-like		N.D.
Lin166B10	-2.25	-1.2 ± 0.3	0.025	0	9e-37	b	LinJ.31.1850	Amino acid permease		N.D.
Lin174F8	-2.11	-1.1 ± 0.1	0.001	0	0	b	LinJ.08.0790	Amastin-like protein		N.D.
Lin188B12	-3.68	-1.9 ± 0.4	0.013	0	0	b	LinJ.31.3400	Sodium stibogluconate resistance protein		N.D.
Lin193E6	-4.16	-2.0 ± 0.3	0.007	0	0	b	LinJ.23.1230	Small hydrophilic endoplasmic reticulum-associated protein (SHERP)		N.D.
Lin202E7	-2.42	-1.3 ± 0.5	0.045	4e-153	0	b	LinJ.31.2060	Succinyl-diaminopimelate desuccinylase-like protein		N.D.
Lin274G6	-2.38	-1.2 ± 0.3	0.020	0	0	b	LinJ.08.0680/90	Amastin-like protein		N.D.
Lin286D1	-2.90	-1.5 ± 0.2	0.011	0	5e-56	b	LinJ.08.1320	Amastin-like protein		N.D.
Lin287H2	-2.17	-1.1 ± 0.3	0.025	0	0	b	LinJ.03.0190	δ-1-pyrroline-5-carboxylate dehydrogenase, putative		N.D.

*The clone aligns with more gene sequences.

### Validation and analysis of unsolved clones by qRT-PCR

Certain clones selected in the microarray hybridization analysis are undetermined because they overlap with more than one gene annotation. Most were solved by TaqMan Probe-based qRT-PCR analyses, thus determining the actual differentially regulated gene. This approach also validated 13.8% of the microarray results (Tables 2 and 3, S4–S7 Tables), together with the internal controls mentioned (S2 Table). The microarray and qRT-PCR results are quite consistent qualitatively. In some cases, remarkable quantitative differences are observed, which is expected due to the inherent wide dynamic range and sensitivity of qRT-PCR compared to the high-throughput microarray hybridization analysis. Constant expression values have been obtained by qRT-PCR in certain clones that overlap with more than one gene. In these cases, at least one of the remaining is presumably differentially regulated. Consequently, inconsistencies between both approaches have not been detected so far.

### Differential gene expression between Pro-Pper and Pro-Stat

**Fig 3:**
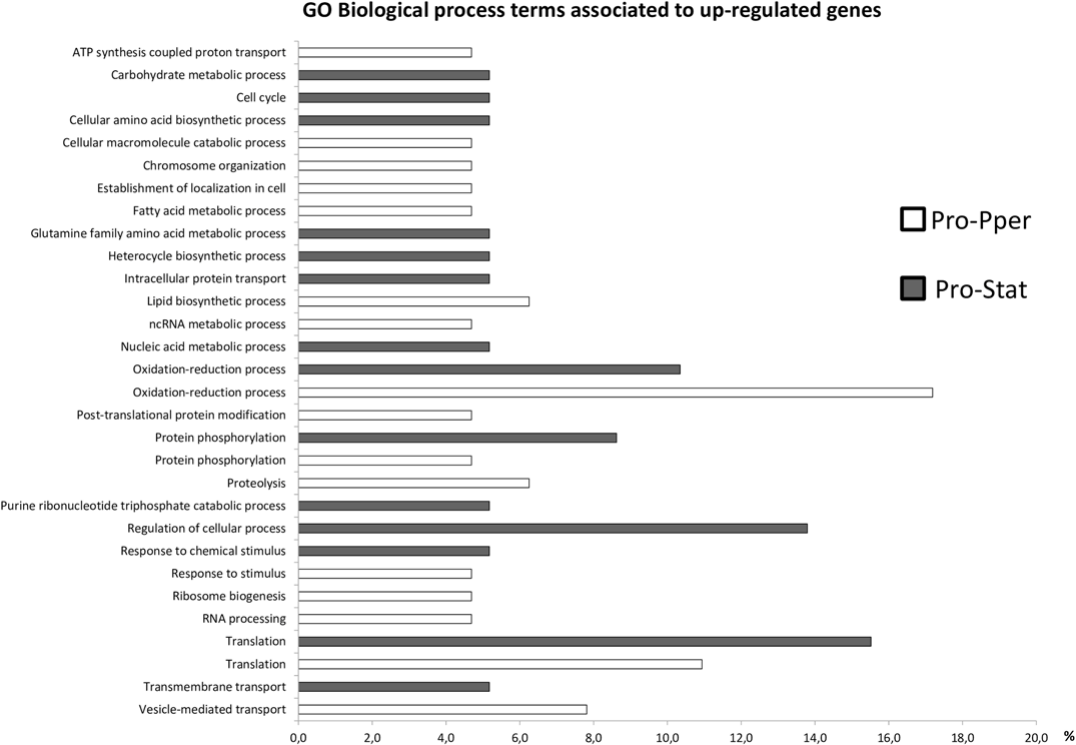
Multi-level bar graph of GO biological function terms annotated in the differentially regulated genes between Pro-Pper and Pro-Stat. The GO terms were assigned to the differentially expressed genes between Pro-Pper and Pro-Stat following the pipeline of the BLAST2GO software. First, the BLAST step was run. Then, the GO term assignment step was performed and terms from the KEGG and the InterPro databases were subsequently assigned, as well as the IUBMB EC identifiers. Then, direct acyclic graphs (DAGs) were generated and multi-level sector graphs generated on the basis of those DAGs were retrieved. Finally, the format of the multi-level sector graphs was changed to bar graphs. Accession numbers of GO terms from top to bottom: GO:0015986; GO:0005975; GO:0007049; GO:0008652; GO:0044265; GO:0051276; GO:0051649; GO:0006631; GO:0009064; GO:0018130; GO:0006886; GO:0008610; GO:0034660; GO:0006259; GO:0055114; GO:0043687; GO:0064681; GO:0006508; GO:0009207; GO:0050794; GO:0042221; GO:0050896; GO:0042254; GO:0006396; GO:0006412; GO:0055085; GO:0016192.

**Fig 4:**
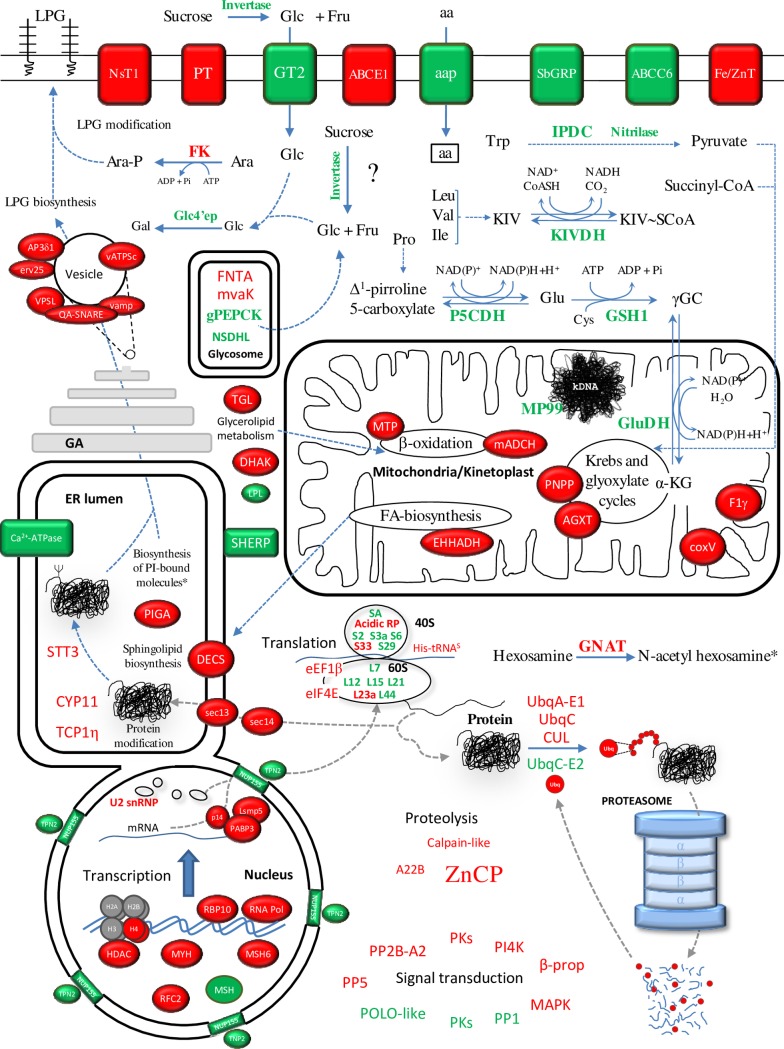
Differential expression profiles of Pro-Pper and Pro-Stat. Protein products in red correspond to genes up-regulated in Pro-Pper and those in green to Pro-Stat. The most relevant changes in transcript relative abundance are discussed in the text. More detailed discussion of all differentially regulated genes is provided in S1 Text. The asterisk indicates that N-acetyl hexosamines are building blocks for PI-bound molecules. Abbreviations not found in the main body of the text: ABC, ATP-binding cassette; AGXT, alanine-glyoxylate transaminase activity; AP3δ1, adaptor complex protein 3 δ subunit 1; coxV, cytochrome oxidase V subunit; CUL, cullin; DECS, sphingolipid δ4-desaturase; DHAK, dihydroxyacetone kinase; EHHADH, 3-hydroxyacyl-ACP dehydratase; erv25, COP-coated vesicle membrane protein erv25; Fe/ZnT, iron/zinc transporter; His-tRNA^S^, histidyl-tRNA synthase; mACDH, mitochondrial acyl-CoA dehydrogenase; MAPK, mitogen-activated protein kinase; MTP, mitochondrial carrier protein; mvaK, mevalonate kinase; NSDHL, NAD(P)-dependent steroid dehydrogenase-like protein; NsT1, nucleoside transporter 1; NUP155, nuclear pore complex protein 155; PNPP, p-nitrophenylphosphatase; PT, pteridine transporter; SbGRP, sodium stibogluconate-resistance protein; TGL, triacylglycerol lipase; TPN2, transportin 2; UbqA-E1, ubiquitin activating enzyme E1; UbqC, ubiquitin conjugating enzyme-like protein; UbqC-E2, ubiquitin conjugating enzyme E2; vamp, vesicle-associated membrane protein; vATPSc, subunit c of the vacuolar ATP synthetase; VPSL, vacuolar protein sorting-like protein.

The GO terms were assigned with BLAST2GO. This software is based on the NCBI database, where the second version of the *L*. *infantum* genome was deposited. In order to ensure that the changes introduced in the last version of the genome sequence released (TriTrypDB) did not affect the GO analysis, we aligned all genes contained in Tables 2 and 3, S4–S7 Tables against the NCBI database. As a result, at least 98% identity was found in all cases. According to the GO analysis, gene expression regulation is affected between Pro-Pper and Pro-Stat. In fact, the GO terms ncRNA metabolic process, RNA processing, translation, post-translational modification and proteolysis are more represented in genes up-regulated in Pro-Pper. Conversely, a considerable number of ribosomal proteins are over-expressed in Pro-Stat (Fig 3). Changes related with signal transduction are also expected because certain differentially expressed protein kinase (PK) and phosphatase (PP) genes are associated to the GO term protein phosphorylation. Some are up-regulated in Pro-Pper and some others in Pro-Stat. All changes in the transcript levels of genes related with gene expression regulation and intracellular signaling might be associated to the GO term response to stimulus (associated to Pro-Pper) and response to chemical stimulus (associated to Pro-Stat) (Fig 3). Certain genes related with vesicle-mediated transport and metabolic processes of carbohydrates, lipids, amino acids and nucleotides are also differentially regulated between Pro-Pper and Pro-Stat. The most relevant differentially regulated genes of known function (Tables 2 and 3) are discussed in detail in this section. A more detailed explanation can be found in S1 Text (S4 and S5 Tables). Important differences in abundance of transcripts related with most major cellular processes have been found between promastigotes developed in both microenvironments studied (Fig 4). Unless indicated, the expression “up-regulation in Pro-Pper” is relative to Pro-Stat and vice versa. The findings described have raised interesting hypotheses that may be tested in the future.

#### DNA repair and regulation of gene expression at the post-transcriptional and post-translational levels

Three genes involved in DNA repair are differentially regulated between Pro-Pper and Pro-Stat: the A/G-specific adenine glycosylase gene (MYH), involved in base excision repair, is up-regulated in Pro-Pper; a mismatch repair protein gene (MSH6) is also up-regulated; and another MSH is down-regulated. Therefore, the MSH6 and MSH genes exhibit opposite expression tendencies in sand fly and in culture

Histone acetylation marks origins of polycistronic transcription in *L*. *major* [36]. Therefore, the up-regulation of the histone deacetylase (HDAC) in Pro-Pper may affect the transcription rate of one or more undetermined PGCs. The up-regulation of a DNA-directed RNA polymerase-like protein (RNA Pol) and a DNA-directed RNA polymerase I subunit (RBP10) may be related to this hypothetical change in histone acetylation somehow. Although the RBP10 subunit is part of the RNA polymerase I complex, it has been predicted to be shared by RNA polymerases I, II and III [37].

At the post-transcriptional regulatory function, certain genes involved in trans-splicing are up-regulated in Pro-Pper, namely the U2 snRNP and the pre-mRNA branch site p14 protein-encoding genes. The poly(A)-binding protein 3 (PABP3) gene, involved in RNA stability, is up-regulated in Pro-Pper (Table 2), as well as in the metacyclic non-agglutinating subpopulation of Pro-Stat as reported [29]. Consequently, higher PABP3 expression levels are found in metacyclic promastigotes in culture and in the sand fly. Provided that the Lsm5p gene has been described to be involved in RNA degradation and processing [38], the up-regulation of this gene in Pro-Pper suggests other unknown hypothetical changes in post-transcriptional regulation processes. Provided that certain translation factors and ribosomal proteins are differentially regulated between Pro-Pper and Pro-Stat, differences in regulation of translation may also exist.

The following levels of gene expression regulation (i.e. protein folding and post-translational modification) may also be influenced by culture according to the up-regulation of related genes in Pro-Pper vs. Pro-Stat. These genes code the translocation complex factors sec13 and sec14 (cytosolic), the subunit 1η of the T complex (Tcp1η), the cyclophilin 11 (CYP11), the UDP-N-acetylglucosamine:phosphatidylinositol α-1,6 N-acetylglucosamine transferase (PIGA), an oligosaccharyl transferase (STT3) acting on asparagine residues, the farnesyltransferase (FNTA) and the prenyl-protein specific carboxymethyltransferase acting on isoprenylcysteine residues (ICMT). The FNTA gene is over-expressed in Pro-Log with respect to Pro-Stat in *L*. *infantum*, whereas de ICMT is down-regulated in Pro-Stat with respect to intracellular amastigotes [28]. The PIGA is involved in the biosynthesis of glycosylphosphatidylinositol (GPI), a component of certain surface glycoproteins, proteophosphoglycans and glycolipids (see below).

Some protease genes are differentially regulated between Pro-Pper and Pro-Stat, including components of the ubiquitin-proteasome degradation system. For example, a Zn-carboxypeptidase gene of the family M14 (ZnCP) is up-regulated in Pro-Pper (Table 2). This gene was also described to be up-regulated in metacyclic promastigotes in axenic culture [29]. The ZnCP may be extracellular, which is supported by TMHMM predictions (S1 Fig). Future studies on this protein may reveal whether it is a good vaccine candidate or pharmacological target.

#### Biosynthesis of surface molecules

The PIGA is involved in the biosynthesis of the GPI, which is part of the lipophosphoglycan (LPG) and the membrane bound proteophosphoglycan (mPPG), and anchors certain glycoproteins. In addition, glycosylinositol phospholipids (GIPLs) are basically free GPI molecules. GIPLs are the major surface molecules in amastigotes and serve as receptors for the host cell and as a shield for resistance against lysosomal hydrolases [39]. The PI4K is involved in the biosynthesis of the PIGA reaction substrate phosphatidylinositol 1-phosphate. The PIGA and the PI4K are up-regulated in Pro-Pper, as well as a glycosyl transferase-like protein gene (GTL), the STT3 and the dolichyl-P-Man:GDP-Man5GlcNAc2-PP-dolichyl α-1,3-mannosyltransferase (ALG3). The ALG3 and the STT3 are involved in N-glycan biosynthesis.

The hydrophilic surface protein (HASPA1) and the small hydrophilic endoplasmic reticulum protein (SHERP) genes are up-regulated in Pro-Stat. Although these molecules are important for development in the sand fly (Pro-Pper), according to this analysis, their relative expression at the transcript level is higher in culture (Pro-Stat). These genes are also over-expressed in Pro-Stat vs. Pro-Log [28]. Provided that Pro-Pper promastigotes were obtained from the anterior part of *P*. *perniciosus* gut at the stomodeal valve (see Methods), promastigotes may increase the expression levels of HASP and SHERP molecules in culture due to the absence of certain environmental signals specific of the sand fly gut microenvironment.

With regard to the amastin superfamily, the genes LinJ.08.0680/0690/0700/0710 and LinJ.34.2660 are up-regulated in Pro-Stat vs. Pro-Pper (Table 3) and vs. Pro-Log [28]. Additionally, LinJ.08.0790/1320 and LinJ.24.1280 are up-regulated in Pro-Stat vs. Pro-Pper. Some of these genes are up-regulated when temperature is raised and pH decreased both in axenic and intracellular amastigotes [17,28]. These molecules may be over-expressed in advance before the differentiation process of promastigotes to amastigotes, which is in agreement with the pre-adaptation hypothesis [28,40].

#### Metabolism

The comparative transcriptome analysis of Pro-Pper and Pro-Stat supports that some genes related with sugar, amino acid and lipid metabolism are differentially expressed (Fig 3, Tables 2 and 3, S4 and S5 Tables). The glucosamine-6-phosphate N-acetyltransferase (GNAT) is up-regulated in Pro-Pper. This gene is also up-regulated in Pro-Log with respect to Pro-Stat in *L*. *infantum* [28] and in Pro-Stat vs. axenic amastigotes obtained by temperature increase plus pH decrease [17] and vs. intracellular amastigotes obtained by *in vitro* infection of the U937 cell line [28]. It has been described that hexosamine phosphates are major carbon sources for *L*. *major* amastigotes; these derivatives accumulate in parasites defective for the glucosamine-6-phospate deaminase [41]. As hexosamines are toxic metabolites produced by the macrophage, catabolism of these molecules is crucial for these parasites [41]. Provided that the GNAT catalyzes reversible transfer of acetyl-CoA to GlcN-6-P yielding GlcNAc-6P, this enzyme may participate in this catabolic process, in biosynthesis of glycoconjugates like the GPI or in the accumulation of GlcN derivatives in *L*. *infantum* Pro-Pper and Pro-Stat [41,42]. In fact, the GNAT gene is also up-regulated in Pro-Pper with respect to intracellular amastigotes [24].

In principle, up-regulation of the invertase in Pro-Stat would somehow suggest a higher degree of sucrose utilization as a source of energy under culture conditions. Indeed, it has been reported that the invertase is secreted to the extracellular milieu [43]. The same may be hypothesized for glucose, given the up-regulation of the glucose transporter 2 (GT2) in Pro-Stat. However, the glycosomal phosphoenolpyruvate carboxykinase gene (gPEPCK) is also up-regulated at this stage. The expression profile of the UDP-glucose 4’ epimerase (Glc4’ep) is also similar, thus suggesting an increased rate of transformation of UDP-Glc into UDP-Gal. These data taken together suggest the utilization of hexoses as building blocks for glycoproteins and phosphoglycans or the pentose-phosphate pathway rather than as carbon and energy sources in Pro-Stat. Particularly, the Gal residues may be used for the biosynthesis of the LPG. The exposed Gal residues of the LPG are blocked by arabinose ones in *L*. *infantum* and *L*. *major* metacyclic promastigotes [44]. Indeed, the fucose kinase (FK) gene, involved in arabinose phosphate biosynthesis [45], is up-regulated in Pro-Pper. GDP-arabinose is the substrate for arabinosyltransferases to transfer arabinose residues to the LPG.

Proline, leucine, isoleucine and valine degradation rates may be increased in Pro-Stat, according to down-regulation of the following genes in Pro-Pper: amino acid permease (AAP), α-ketoisovalerate dehydrogenase LinJ.23.0620 (α-KIVDH), δ-1-pyrroline-5-carboxylate dehydrogenase (P5CDH), pyrroline-5-carboxylate reductase (PC5R) and glutamate dehydrogenase (GDH). In addition, indoleacetate generation might be less active in Pro-Pper in light of the down-regulation of the α-ketoacid decarboxylase gene (IPDC), which bears the pyruvate (EC 4.1.1.1.) and indolepyruvate decarboxylase (EC.4.1.1.74.) activities. The nitrilase (EC 3.5.5.1) is also involved in tryptophan metabolism and its steady-state levels are lower in Pro-Stat. Most tryptophan degradation enzymes are not present in *Leishmania* spp. (KEGG database). However, the IPDC gene is annotated. Indeed, most aromatic amino acid oxidation pathways are missing in the parasite and intermediate products are excreted [46]. Lysine synthesis may be less active in Pro-Pper because the succinyl-diaminopimelate desuccinylase gene (DAPDS) is down-regulated. Glutathione biosynthesis may be higher under stationary phase culture conditions as well, as the γ-glutamylcysteine synthase (GSH1) is up-regulated in Pro-Stat. Taken as a whole, these data suggest that the major carbon and energy sources of Pro-Stat are amino acids. A different scenario is observed in Pro-Pper, where genes related to lipid metabolic processes are up-regulated (Figs 3 and 4, Table 2; S4 Table). Detailed discussion of these data is provided in S1 Text, suggesting that different lipid catabolic and anabolic processes may take place between Pro-Pper and Pro-Stat.

#### Redox homeostasis

Unlike *L*.*infantum* cultured promastigotes, the motile choanomastigote stage of the monogenetic trypanosomatid *Crithidia fasciculata* over-expresses the trypanothione peroxidase (TryP). This is presumably due to a differentiation process in culture [47]. TryP is not differentially regulated in *L*. *infantum* promastigotes within the sand fly anterior midgut, which has been confirmed by qRT-PCR (Table 2). It is not differentially regulated between Pro-Log and Pro-Stat either [28].

#### Transporters and cytoskeleton

Certain genes involved in ATP synthesis coupled to proton transport, transmembrane and vesicle-mediated transport are differentially regulated between Pro-Pper and Pro-Stat (Figs 3 and 4). Others are involved in organization of the actin and tubulin cytoskeleton components, including genes encoding actin, actin-interacting protein (AIP), tubulin, kinesin and dynein genes. Detailed discussion about these changes is included in S1 Text.

#### Intracellular signaling

**Fig 5:**
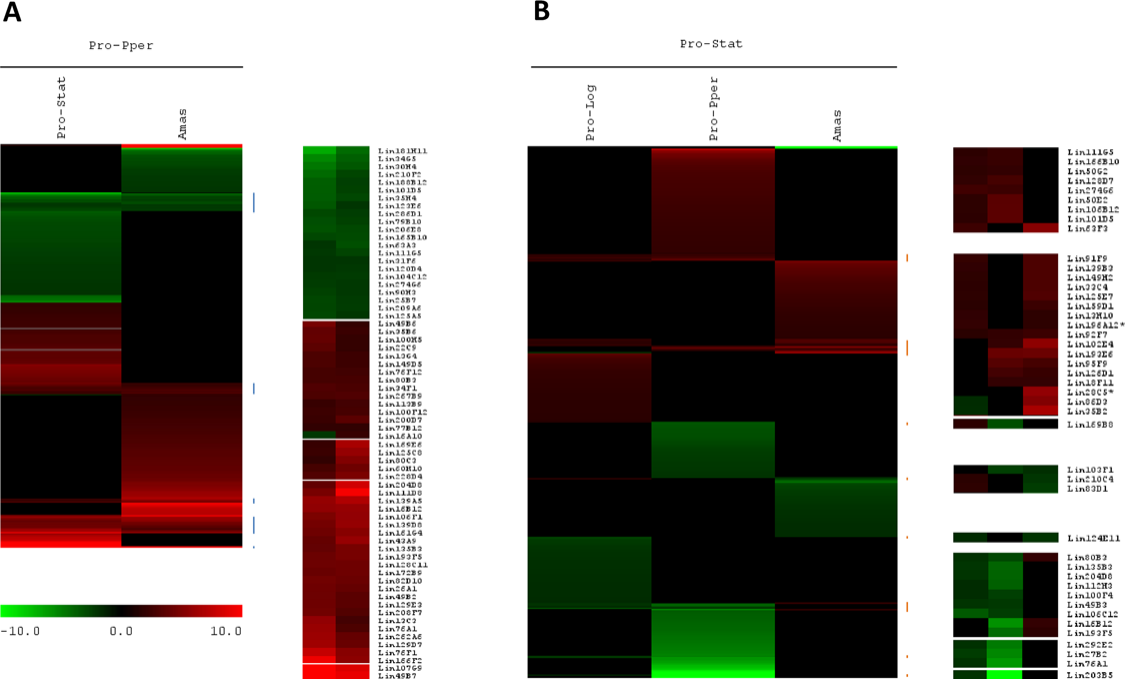
HCL-ST comparison of Pro-Pper with cultured promastigotes and amastigotes of *L*. *infantum*. (A) Pro-Pper vs. Pro-Stat (this study) and Amas [24]. (B) Pro-Stat vs. Pro-Pper (this study), Pro-Log and Amas [28]. A description of clones not found in S4–S8 Tables can be found in [28]. All the microarray hybridization experiments were performed by the same procedure and clone nomenclature is equivalent (see Availability of the Supporting Data). For obvious reasons, when more than one gene is represented by a given clone, independent qRT-PCR analysis was performed. This approach allows determining the actual gene that is differentially expressed in each different biological condition (Tables 2 and 3, S4 and S5 Tables [24,28]).

Despite the kinome of trypanosomatids has been well characterized [48], signal transduction pathways have not been elucidated in these organisms yet [49]. These pathways are probably different to those found in model organisms like yeasts. In addition, the connections between external stimuli, the transduction mechanisms and the expression regulation processes of the target gene are unknown in trypanosomatids so far. However, information about differential expression of genes encoding signaling molecules is available (e.g., [28,50]), including this study. Indeed, some genes involved in signaling processes are differentially regulated between Pro-Pper and Pro-Stat (Fig 4). Most are related with protein phosphorylation (Fig 3). Not surprisingly, a few protein kinases and phosphatases are up-regulated in Pro-Pper whereas others are down-regulated. In fact, the genome of the parasite encodes a repertoire of about 200 protein kinases [51] that may have different biological roles depending on the physiological context of the parasite. Other signaling genes are differentially regulated between Pro-Pper and Amas [24]. As discussed below, four of them are common between the Pro-Pper/Pro-Stat and the Pro-Pper/Amas profiles (Fig 5A). Future elucidation of signaling pathways might contribute to explain connections between environmental stimuli and the parasite's response for survival and development.

### Gene clustering supports crucial changes in Pro-Pper gene expression for life cycle progression

The differential expression profile of Pro-Pper/Pro-Stat described in this study has been compared to the Pro-Pper/Amas [24], the Pro-Stat/Pro-Log and the Pro-Stat/Amas [28] expression profiles by HCL-ST (Fig 5). All microarray hybridization experiments were performed by the same procedure and clone nomenclature is equivalent. Fig 5A consists of clusters obtained by HCL-ST of two sets of differentially regulated genes: Pro-Pper/Pro-Stat (286 genes; Tables 2 and 3, S4–S8 Tables) and Pro-Pper/Amas (213 genes [24]). The intersection of both sets is 64 genes (30.0% and 22.4%, respectively). Two thirds of these 64 common changes (20.2% and 15.0%, respectively) correspond to up-regulated genes in Pro-Pper and the remaining 21 (9.9% and 7.3%, respectively) to down-regulated. In principle, this suggests that common over-expression of the 43 genes in Pro-Pper vs. Pro-Stat and vs. Amas is important for life cycle progression, i.e. for development of promastigotes from the sand fly anterior thoracic midgut into amastigotes. In other words, up-regulation of these genes is required at the beginning of differentiation into amastigotes only whether promastigotes have been developed within the sand fly gut but not in axenic culture. In fact, none of these genes is up-regulated in Pro-Stat vs. Amas (Fig 5B) [28]. Conversely, the 21 commonly down-regulated genes in Pro-Pper vs. Pro-Stat and vs. Amas indicate that Pro-Stat require higher steady-state levels of the corresponding transcripts upon differentiation to amastigotes.

Differentiation of promastigotes to an infective stage is successful in culture, as well as within the sand fly gut. However, the efficiency of infection is higher in the natural life cycle than in experimental infections of cell lines and animals. For example, a single *P*. *dubosqi* sand fly is able to transmit as low as 600–1,000 *L*. *major* promastigotes capable of establishing infection in mice successfully [52], whereas experimental infection with *L*. *major* stationary phase promastigotes is usually performed with 10^6^ promastigotes (e.g. [53]). This may be explained by the *in vitro* infection procedure, the reduction of infectivity of cultured parasites [14] and the absence of molecules from the saliva of the sand fly. In fact, *L*. *infantum* Pro-Pper promastigotes are significantly and considerably more infective than Pro-Stat (Fig 1B). Therefore, future studies on the 43 commonly up-regulated genes in Pro-Pper vs. Pro-Stat and vs. Amas may contribute to explain the increased infection rate during progression of the natural life cycle.

Lahav et al. [54] described that the correlation between the transcript and protein levels is about 25% quantitatively in *L*. *donovani*. According to clustering analysis, qualitative coincidence (up-regulation, down-regulation, constitutive expression) is about 65% [54]. Proteome analysis is not possible in Pro-Pper due to sample amount limitations. Therefore, amplification of the transcriptome is the only alternative so far. For this reason, our estimate of genes that are actually related with progression of the life cycle is at least 29 in sand fly foregut promastigotes and 13 in stationary phase promastigotes. Future studies on these genes may reveal new vaccine candidates and drug targets. For example, we described that the *L*. *infantum* tyrosine aminotransferase is up-regulated in metacyclic promastigotes in culture at the transcript level [29] and it has been confirmed at the protein level by Western blot. This protein is a drug target candidate and the pharmacophore has been predicted [55] after structural study [56].

GO terms were associated to differentially regulated genes in Pro-Pper/Pro-Stat (Fig 3) and Pro-Pper/Amas [24]. According to this analysis, genes involved in carbohydrate and amino acid metabolic processes are up-regulated in Pro-Stat and Amas with respect to Pro-Pper, whereas cellular macromolecule catabolic processes, fatty acid metabolic processes and vesicle mediated transport are linked to genes up-regulated in Pro-Pper. In both comparisons, changes in steady-state transcript levels involved in response to internal and external stimuli were found to be higher in Pro-Pper, except for response to chemical stimuli. In the case of the Pro-Pper/Amas comparison, the systematic analysis of GO terms provided insight into the nature of the stimuli (abiotic stimuli, DNA damage and inorganic substances) [24], unlike in Pro-Pper/Pro-Stat. In both cases, the considerable number of differences in genes related to intracellular signaling and regulation of gene expression is remarkable, including the post-transcriptional, translational and post-translational levels and protein degradation via the ubiquitin-proteasome system. In fact, the 43 commonly up-regulated genes in Pro-Pper vs. Pro-Stat and vs. Amas are involved in most major cellular processes like DNA repair (MSH6), gene expression regulation (H4, HDAC, U2-snRNP, Lsm5p, eIF4E, 60S acidic ribosomal protein), proteolysis (UBC, ubq, A22B), cytoskeleton remodeling (PFN), intracellular signaling (PP2B-A2, PK, β-prop, PI4K), metabolism (GNAT, DHAK, coxV, oxidoreductase), transport (PT, ABCE1, AP3δ1) and biosynthesis of surface molecules (PI4K, PIGA). Nineteen hypothetical proteins are also included. Among these genes, H4, GNAT, AP3δ1 and four hypothetical protein genes are also up-regulated in Pro-Log and the expression levels of GNAT and PP2B-A2 are higher in Pro-Stat than in Amas (Fig 5B) [28]. The 21 up-regulated genes in Pro-Stat and Amas with respect to Pro-Pper are ESAG5, L21, gPEPCK, NSDHL, SbGRP, AIP, three amastins and 12 hypothetical protein genes. Three of them (gPEPCK and amastin-like proteins LinJ.08.0680/90) are also over-expressed in Pro-Stat with respect to Pro-Log (Fig 5B) [28]. Future studies on these genes that are common to both datasets are of great interest. The first set may contribute to explain the relationship between signal transduction and effector mechanisms of gene expression regulation in the differentiation process of Pro-Pper to Amas, whereas the second set would be applicable for the equivalent process from Pro-Stat to Amas.

In summary, most differential gene expression profiles are distinct between Pro-Pper/Pro-Stat and Pro-Pper/Amas as expected, except for 13.2% genes specifically up-regulated by the effect of the microenvironment (i.e. sand fly gut or culture) at the beginning of differentiation of promastigotes to amastigotes. Therefore, these genes are essential for life cycle progression in the respective microenvironments. In fact, they participate in processes affecting key regulatory biological processes.

### Is axenic culture of promastigotes an acceptable model?

The data presented above contribute to answer this question. Axenic cultures of promastigotes mimic the conditions inside the gut of the sand fly to some extent [10,11,12,13] and they are relatively stable and reproducible when compared with amastigote cultures [16,17]. However, this study confirms that *L*. *infantum* promastigotes obtained from the anterior thoracic midgut of *P*. *perniciosus* are considerably more infective than promastigotes in stationary phase of axenic culture (Fig 1B). Evidence about important differences in their transcriptome is also provided, i.e. 286 differentially regulated genes. As described above, the correlation coefficient between both expression datasets is R^2^ = 0.727 (Fig 2). The meaning of this finding is that the Pro-Pper and Pro-Stat populations are strongly correlated, although important differences are still observed. Indeed, the shape of the M/A scatter plot (Fig 2) is a non-dispersed (rank -4 < M < 4) dot-cloud symmetric about the M = 0 line (i.e., lack of differential expression). In light of these findings, we contend that the axenic culture model of promastigotes is generally valid, but it should be cautiously questioned case by case for every particular experimental design. After all, Pro-Pper promastigotes are the result of development in their natural microenvironment and consequently, their infectivity is higher.

### Conclusions

Pro-Pper populations are more infective than Pro-Stat ones. Their transcriptome profiles are substantially different. In fact, certain genes involved in DNA repair, gene expression regulation, metabolism, transport including vesicle trafficking, intracellular signaling, cytoskeleton remodeling and biosynthesis of surface molecules are differentially regulated. However, the Pearson correlation coefficient between the normalized fluorescence intensity values of both populations is R^2^ = 0.727. This indicates strong correlation but also remarkable differences. Consequently, the adequacy of the axenic culture model should be studied *a priori* in each particular experimental design.

The HCL-ST analysis has revealed that the degree of similarity in differentially expressed genes between Pro-Pper/Pro-Stat and Pro-Pper/Amas is 13.2% (64 genes). Life cycle progression (differentiation to amastigote) in the natural microenvironment of promastigotes would require up-regulation of 43 out of the 64 common genes. The information obtained in this high-throughput study is useful for understanding better the differences between promastigotes from culture and the sand fly. Specific information about relative expression is also a criterion for selecting possible vaccine candidates and/or drug targets.

## Supporting Information

S1 Fig ZnCP transmembrane domain prediction with TMHMM. (PPT)

S1 Table Primers and TaqMan-MGB probes used in qRT-PCR validation and determination of differential expression in unresolved clones. (XLS)

S2 Table Microarray controls. Results of the Pro-Per/Pro-Stat cDNA-genomic DNA microarray hybridization analysis for positive and negative control spots.
(DOC)

S3 Table Mean fluorescence intensity values. Mean fluorescence intensity values obtained in Pro-Per/Pro-Stat cDNA-genomic DNA microarray hybridization.
(XLS)

S4 Table Complete list of up-regulated genes of known function in Pro-Pper. (DOC)

S5 Table Complete list of up-regulated genes of known function in Pro-Stat. (DOC)

S6 Table Hypothetical protein genes up-regulated in Pro-Pper/Pro-Stat. (DOC)

S7 Table Hypothetical protein genes down-regulated in Pro-Pper/Pro-Stat. (DOC)

S8 Table Type c and qRT-PCR undetermined clones. (DOC)

S1 Text Detailed discussion of the differential gene expression profiles between Pro-Pper and Pro-Stat. (DOC)
