# Supplementary material for: Influence of the Microenvironment in the Transcriptome of Leishmania infantum Promastigotes: Sand Fly versus Culture
Source: PLoS Negl Trop Dis. 2016 May 10;10(5):e0004693. doi: 10.1371/journal.pntd.0004693 (PMC4862625; doi:10.1371/journal.pntd.0004693)
Supplement: S1 Fig — (PPT) [file pntd.0004693.s001.ppt]

## Slide 1
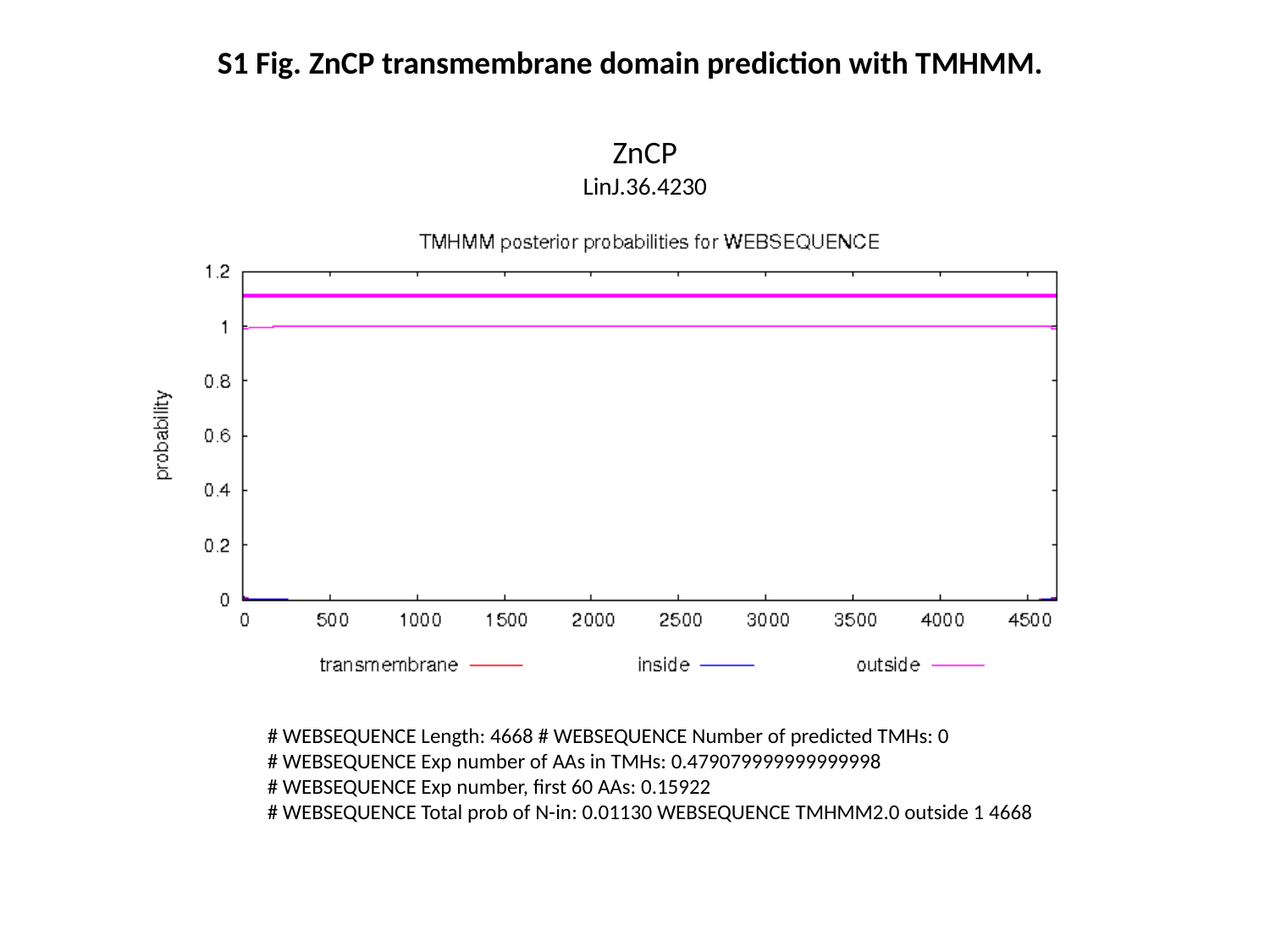

S1 Fig. ZnCP transmembrane domain prediction with TMHMM.
ZnCP
LinJ.36.4230
# WEBSEQUENCE Length: 4668 # WEBSEQUENCE Number of predicted TMHs: 0
# WEBSEQUENCE Exp number of AAs in TMHs: 0.479079999999999998
# WEBSEQUENCE Exp number, first 60 AAs: 0.15922
# WEBSEQUENCE Total prob of N-in: 0.01130 WEBSEQUENCE TMHMM2.0 outside 1 4668
