# Supplementary material for: Influence of the Microenvironment in the Transcriptome of Leishmania infantum Promastigotes: Sand Fly versus Culture
Source: PLoS Negl Trop Dis. 2016 May 10;10(5):e0004693. doi: 10.1371/journal.pntd.0004693 (PMC4862625; doi:10.1371/journal.pntd.0004693)
Supplement: S2 Table — Results of the Pro-Per/Pro-Stat cDNA-genomic DNA microarray hybridization analysis for positive and negative control spots. (DOC) [file pntd.0004693.s003.doc]

##### S2 Table. Microarray controls. Results of the Pro-Per/Pro-Stat cDNA-genomic DNA microarray hybridization analysis for positive and negative control spots. Pro-Pper/Pro-Stat fold changes (F) and standard deviations (SD) are detailed, as well as p-value, (α = 0.05). Absence of differential gene expression has been observed in positive controls (*p* ≥ 0.05). Mean fluorescence intensity (FI) and the associated SD values are provided.

| ***Spot*** | | **F ± SD** | | ***p*** | | | | **Positive control** | |
| --- | --- | --- | --- | --- | --- | --- | --- | --- | --- |
| cLin79A1 | | -1.3 ± 0.6 | 0.120 | | | | *Li Polβ* | | |
| cLin79A2 | | 1.1 ± 1.0 | 0.229 | | | | *Li TopoII* | | |
| cLin79A3 | | 1.2 ± 0.4 | 0.060 | | | | *Li p36* | | |
| cLin79B1 | | 1.0 ± 0.1 | 0.448 | | | | *Li hsp70* | | |
| cLin79B2 | | -1.1 ± 0.2 | 0.296 | | | | *Ldo hsp70* | | |
| cLin79B3 | | -1.2 ± 0.3 | 0.475 | | | | *Lam hsp70* | | |
| cLin79C1 | | 1.1 ± 0.1 | 0.367 | | | | *Lma hsp70* | | |
| cLin79C2 | | 1.0 ± 0.0 | 0.313 | | | | *Li A2* | | |
| cLin79C3 | | -1.1 ± 0.2 | 0.477 | | | | *Ldo A2* | | |
| cLin79D1 | | 1.1 ± 0.1 | 0.410 | | | | *Li GAPDH* | | |
| cLin79D2 | | 1.1 ± 0.1 | 0.741 | | | | *Ldo GAPDH* | | |
| cLin79D3 | | 1.1 ± 0.1 | 0.605 | | | | *LigDNA* | | |
| cLin79H2 | | 1.1 ± 0.2 | 0.547 | | | | *Herring sperm DNA* | | |
| ***Spot*** | **Mean FI ± SD** | | | | **Negative control** | | | |  |
| cLin79E1 | | 583± 230 | | | | *Lfe nifA/hlyD* | | | |
| cLin79E2 | | 224 ± 326 | | | | *Lfe nifD/nifK* | | | |
| cLin79E3 | | 408 ± 37 | | | | *Lfe nifH* | | | |
| cLin79F1 | | 270 ± 110 | | | | *Lfe nifS/nifU* | | | |
| cLin79F2 | | 216 ± 49 | | | | *Lfe nifX/nifB* | | | |
| cLin79F3 | | 120 ± 54 | | | | *Lfe nifH/nifD* | | | |
| cLin79G1 | | 153 ± 25 | | | | *Lfe nifE* | | | |
| cLin79G2 | | 111 ± 81 | | | | *Lfe nifV/HesB* | | | |
| cLin79G3 | | 31 ± 2 | | | | *Lfe nifV* | | | |
| cLin79H1 | | 108 ± 62 | | | | *Lfe nifW/Bgene* | | | |
| cLin79H3 | | 30 ± 9 | | | | *1XSSC* | | | |
|  | |  | | | | | |  | |
