# Supplementary material for: Influence of the Microenvironment in the Transcriptome of Leishmania infantum Promastigotes: Sand Fly versus Culture
Source: PLoS Negl Trop Dis. 2016 May 10;10(5):e0004693. doi: 10.1371/journal.pntd.0004693 (PMC4862625; doi:10.1371/journal.pntd.0004693)
Supplement: S4 Table — (DOC) [file pntd.0004693.s005.doc]

**S4 Table. Complete list of up-regulated genes of known function in Pro-Pper.** The following items are specified for each selected clone: fold change (F ≥ 2); SD; Student’s t-test p-value; expect value in alignments (e-value); clone definition according to mapping outcomes a, b and c; Gene Id. retrieved from the database TriTrypDB; annotated functions in the *L. infantum* genome sequence; qRT-PCR outcomes. See more detailed information in the Methods section.

| ***Clone*** | ***F*** | ***log_2_F ± SD*** | ***p*** | ***e-value*** | | ***Def.*** | ***Gene Id. (TriTrypDB)*** | ***Annotated gene function*** | ***qRT-PCR*** | |
| --- | --- | --- | --- | --- | --- | --- | --- | --- | --- | --- |
|  |  |  |  | ***Fw*** | ***Rv*** |  |  |  |  |  |
| Lin9E5 | 4.24 | 2.1 ± 0.1 | 0.001 | 0 | 0 | b | LinJ.35.1150 | Oligosaccharyl transferase-like protein |  | N.D. |
| Lin10H12 | 2.78 | 1.5 ± 0.1 | 0.003 | 0 | 3e-132 | b | LinJ.31.0860 | Lipase |  | N.D. |
|  |  |  |  |  |  |  | LinJ.31.0870 | Lipase precursor-like protein |  | N.D. |
| Lin12D2 | 3.32 | 1.7 ± 0.4 | 0.016 | 0 | 0 | b | LinJ.24.1890 | Short chain dehydrogenase/reductase, putative | + | 2.2 ± 0.2 |
|  |  |  |  |  |  |  | LinJ.24.1900 | Hypothetical protein, conserved |  | N.D. |
| Lin13C3 | 5.77 | 2.5 ± 1.0 | 0.049 | 3e-175 | 0 | b | LinJ.21.0770 | ATP-binding cassette protein subfamily E, member 1, putative (ABCE1) |  | N.D. |
| Lin13G4 | 3.12 | 1.6 ± 0.1 | 0.002 | 0 | 0 | c | LinJ.29.0990 | Signal peptide peptidase, putative, aspartic peptidase, Clan AD, family A22B, putative | + | 2.5 ± 0.1 |
| Lin16C12 | 14.35 | 3.8 ± 0.6 | 0.009 | 0 | 0 | b | LinJ.35.3930 | Calmodulin-like protein, EF hand-containing protein | + | 653.3 ± 20.6 |
|  |  |  |  |  |  |  | LinJ.35.3940 | Hypothetical protein, conserved |  | N.D. |
| Lin21H10 | 2.13 | 1.1 ± 0.4 | 0.038 | 0 | 0 | b | LinJ.26.1670 | Sphingolipid δ4 desaturase, putative | + | 2.1 ±0.1 |
| Lin22B1 | 3.76 | 1.9 ± 0.2 | 0.005 | 0 | 0 | a | LinJ.23.1390 | Hypothetical protein, conserved |  | N.D. |
|  |  |  |  |  |  |  | LinJ.23.1400 | Coronin (CRN12) | + | 5.3 ± 0.4 |
| Lin22C9 | 3.75 | 1.9 ± 0.4 | 0.018 | 0 | 0 | b | LinJ.33.2910 | Ubiquitin-conjugating enzyme, putative |  | N.D. |
| Lin26A9 | 6.18 | 2.6 ± 0.5 | 0.011 | 0 | 0 | a | LinJ.26.2290 | Nitrilase, putative | + | 2.7 ± 0.3 |
|  |  |  |  |  |  |  | LinJ.26.2300 | Hypothetical protein, conserved |  | N.D. |
|  |  |  |  |  |  |  | LinJ.26.2310 | Hypothetical protein, conserved |  | N.D. |
| Lin27B2 | 5.28 | 2.4 ± 0.6 | 0.023 | 0 | 0 | b | LinJ.35.1230 | Short chain dehydrogenase, putative |  | N.D. |
|  |  |  |  |  |  |  | LinJ.35.1240 | Short chain dehydrogenase, putative |  | N.D. |
| Lin28B4 | 2.18 | 1.1 ± 0.1 | 0.002 | 8e-142 | 0 | b | LinJ.26.1620 | 40S ribosomal protein S33, putative | + | 9.9 ± 0.8 |
|  |  |  |  |  |  |  | LinJ.26.1630 | 40S ribosomal protein S33, putative | + | 9.9 ± 0.8 |
|  |  |  |  |  |  |  | LinJ.26.1640 | Hypothetical protein, conserved |  | N.D. |
| Lin28B5 | 4.72 | 2.2 ± 0.5 | 0.014 | 0 | - | c | LinJ.35.1230 | Short chain dehydrogenase, putative |  | N.D. |
| Lin31D4 | 3.22 | 1.7 ± 0.2 | 0.004 | 5e-131 | 0 | b | LinJ.36.4230 | Zinc carboxypeptidase, putative, metallo-peptidase, Clan MC, family M14 |  | N.D. |
| Lin32A10 | 2.46 | 1.3 ± 0.4 | 0.038 | 0 | 0 | b | LinJ.32.3080 | Tubuline-tyrosine ligase-like protein |  | N.D. |
| Lin34F1 | 3.20 | 1.7 ± 0.4 | 0.017 | 0 | 0 | b | LinJ.08.1000 | Histone deacetilase, putative |  | N.D. |
| Lin41D1 | 3.55 | 1.8 ± 0.1 | 0.001 | 0 | 0 | c | LinJ.23.0060 | Cyclophilin 11, putative (CYP11) |  | N.D. |
| Lin44C6 | 11.88 | 3.6 ± 1.2 | 0.034 | 0 | 0 | a | LinJ.31.3310 | Hypothetical protein, conserved |  | N.D. |
|  |  |  |  |  |  |  | LinJ.31.3320 | Histone H4, putative |  | N.D. |
| Lin49B3 | 2.27 | 1.2 ± 0.1 | 0.003 | 0 | 0 | a | LinJ.31.0860 | Lipase |  | N.D. |
|  |  |  |  |  |  |  | LinJ.31.0870 | Lipase precursor-like protein |  | N.D. |
| Lin49B6 | 4.72 | 2.2 ± 0.3 | 0.008 | 0 | 0 | b | LinJ.06.1310 | Hypothetical protein, conserved |  | N.D. |
|  |  |  |  |  |  |  | LinJ.06.1320 | Pteridine transporter, putative |  | N.D. |
| Lin49B7 | 15.89 | 4.0 ± 0.5 | 0.006 | 0 | 0 | b | LinJ.36.2050 | Mismatch repair protein MSH8, putative |  | N.D. |
| Lin59H2 | 3.10 | 1.6 ± 0.1 | 0.001 | 6e-115 | 2e-87 | b | LinJ.23.0040 | β-propeller protein, putative | + | 5.0 ± 0.2 |
|  |  |  |  |  |  |  | LinJ.23.0050 | Peroxidoxin (Tryparedoxin peroxidase) | **-** | 1.3 ± 0.1 |
|  |  |  |  |  |  |  | LinJ.23.0060 | Cyclophilin 11, putative (CYP11) | + | 9.1 ± 0.6 |
| Lin60C1 | 2.17 | 1.1 ± 0.1 | 0.002 | 0 | 0 | a | LinJ.18.0150 | Serine/Threonine protein phosphatase type 5, putative | + | 10.4 ± 0.3 |
|  |  |  |  |  |  |  | LinJ.18.0160 | Hypothetical protein, conserved |  | N.D. |
| Lin60H10 | 2.79 | 1.5 ± 0.3 | 0.013 | 0 | 0 | a | LinJ.23.0630 | Oxidoreductase-like protein | + | 2.4 ± 0.0 |
|  |  |  |  |  |  |  | LinJ.23.0640 | Hypothetical protein, conserved |  | N.D. |
| Lin64A8 | 2.88 | 1.5 ± 0.1 | 0.003 | 0 | 0 | b | LinJ.36.3570 | Short chain dehydrogenase-like protein |  | N.D. |
| Lin72A2 | 2.16 | 1.1 ± 0.2 | 0.009 | 0 | 0 | b | LinJ.36.0640 | Sec14, cytosolic factor |  | N.D. |
| Lin76A1 | 6.02 | 2.6 ± 0.8 | 0.028 | 0 | 0 | b | LinJ.31.3320 | Histone H4, putative |  | N.D. |
| Lin76F1 | 7.81 | 3.0 ± 1.1 | 0.044 | 0 | 0 | b | LinJ.34.3370 | Phosphatidylinositol 4-kinase, putative |  | N.D. |
| Lin76F12 | 2.76 | 1.5 ± 0.2 | 0.006 | 0 | 0 | c | LinJ.10.0430 | Dihydroxyacetone kinase 1-like protein | + | 2.0 ± 0.1 |
| Lin77B12 | 2.03 | 1.0 ± 0.1 | 0.002 | 0 | 0 | b | LinJ.27.1520 | Eukaryotic translation initiation factor eIF4E, putative |  | N.D. |
| Lin79E10 | 2.00 | 1.0 ± 0.2 | 0.011 | 0 | 0 | c | LinJ.04.0740 | Hypothetical protein, conserved |  | N.D. |
|  |  |  |  |  |  |  | LinJ.36.3860 | Calmodulin, putative | + | 2.3 ± 0.1 |
| Lin80B3 | 2.79 | 1.5 ± 0.2 | 0.005 | 0 | 0 | b | LinJ.28.3250 | Glucosamine-6-phosphate N-acetyltransferase, putative |  | N.D. |
| Lin80C3 | 2.00 | 1.0 ± 0.2 | 0.011 | 0 | 0 | b | LinJ.28.3250 | Glucosamine-6-phosphate N-acetyltransferase, putative |  | N.D. |
| Lin82D10 | 4.51 | 2.2 ± 0.4 | 0.010 | 0 | 0 | a | LinJ.23.0040 | β-propeller protein, putative | + | 5.0 ± 0.2 |
|  |  |  |  |  |  |  | LinJ.23.0050 | Peroxidoxin (Tryparedoxin peroxidase) | **-** | 1.3 ± 0.1 |
|  |  |  |  |  |  |  | LinJ.23.0060 | Cyclophilin 11, putative (CYP11) | + | 9.1 ± 0.6 |
| Lin83C12 | 2.76 | 1.5 ± 0.4 | 0.020 | 0 | 0 | b | LinJ.36.2160 | Dolichyl-P-Man:GDP-Man5GlcNAc2-PP-dolichyl α-1,3-mannosyltransferase, putative (ALG3) |  | N.D. |
| Lin100E10 | 2.17 | 1.1 ± 0.2 | 0.013 | 2e-40 | 0 | b | LinJ.35.3900 | T-complex protein 1, η subunit, putative |  | N.D. |
| Lin100F4 | 2.48 | 1.3 ± 0.5 | 0.049 | 0 | 0 | a | LinJ.28.2280 | Dynein light chain LC6, flagellar outer arm, putative | + | 16.8 ± 0.9 |
|  |  |  |  |  |  |  | LinJ.28.2290 | A/G-specific adenine glycosylase, putative | - | 2.0 ± 0.0 |
| Lin100F12 | 2.48 | 1.3 ± 0.5 | 0.049 | 0 | 0 | a | LinJ.30.3040 | Lsm5p, putative | + | 3.4 ± 0.2 |
|  |  |  |  |  |  |  | LinJ.30.3050 | Hypothetical protein, conserved |  | N.D. |
|  |  |  |  |  |  |  | LinJ.30.3030 | Hypothetical protein, conserved |  | N.D. |
| Lin102G11 | 2.60 | 1.4 ± 0.5 | 0.045 | 0 | 0 | b | LinJ.13.1020 | DNA-directed RNA-polymerase I subunit, putative, RNA binding protein (RBP10), putative | + | 14.2 ± 0.7 |
|  |  |  |  |  |  |  | LinJ.13.1030 | Hypothetical protein, conserved |  | N.D. |
|  |  |  |  |  |  |  | LinJ.13.1040 | Hypothetical protein, conserved |  | N.D. |
| Lin103B4 | 2.47 | 1.3 ± 0.1 | 0.005 | 0 | 0 | a | LinJ.30.2620 | Replication factor c, subunit 2, putative | + | 2.1 ± 0.1 |
|  |  |  |  |  |  |  | LinJ.30.2630 | Hypothetical protein, conserved |  | N.D. |
| Lin103E2 | 3.16 | 1.7 ± 0.2 | 0.004 | 0 | 0 | b | LinJ.31.3180 | Fe/Zn transporter protein-like protein |  | N.D. |
| Lin103F1 | 2.48 | 1.3 ± 0.2 | 0.008 | 0 | 0 | a | LinJ.35.3080 | Prenyl protein-specific carboxymethyltransferase, putative |  | N.D. |
| Lin106C5 | 2.65 | 1.4 ± 0.2 | 0.007 | 0 | 0 | b | LinJ.23.0030 | Hypothetical protein, conserved |  | N.D. |
|  |  |  |  |  |  |  | LinJ.23.0040 | β-propeller protein, putative | + | 5.0 ± 0.2 |
|  |  |  |  |  |  |  | LinJ.23.0050 | Peroxidoxin (Tryparedoxin peroxidase) | **-** | 1.3 ± 0.1 |
| Lin106C12 | 2.42 | 1.3 ± 0.3 | 0.023 | 0 | 0 | b | LinJ.08.0010 | Adaptor complex protein (AP) 3δ subunit 1, putative | + | 9.1 ± 0.6 |
|  |  |  |  |  |  |  | LinJ.08.0020 | Hypothetical protein, conserved |  | N.D. |
|  |  |  |  |  |  |  | LinJ.08.0030 | Vesicle-associated membrane protein, putative | + | 7.1 ± 0.3 |
| Lin107C2 | 2.29 | 1.2 ± 0.1 | 0.004 | 0 | 0 | b | LinJ.08.1000 | Histone deacetilase, putative |  | N.D. |
| Lin111D8 | 4.80 | 2.3 ± 0.3 | 0.006 | 0 | 0 | a | LinJ.08.1000 | Histone deacetilase, putative |  | N.D. |
| Lin112A4 | 2.49 | 1.3 ± 0.4 | 0.030 | 1e-69 | 5e-72 | b | LinJ.32.0050 | Protein transport protein sec13, putative |  | N.D. |
| Lin112H3 | 3.79 | 1.9 ± 0.6 | 0.029 | 0 | 0 | a | LinJ.29.2070 | Protein farnesyltransferase α subunit, putative |  | N.D. |
| Lin112H8 | 3.24 | 1.6 ± 0.4 | 0.008 | 8e-170 | 0 | b | LinJ.28.2270 | Cullin 2, putative |  | N.D. |
| Lin113B9 | 2.20 | 1.1 ± 0.1 | 0.006 | 0 | 0 | b | LinJ.36.0550 | Hypothetical protein, conserved |  | N.D. |
|  |  |  |  |  |  |  | LinJ.36.0570 | U2 small nuclear ribonucleoprotein 16.5K, putative | + | 10.4 ± 1.0 |
| Lin123G3 | 9.84 | 3.3 ± 0.4 | 0.006 | 0 | 0 | a | LinJ.23.0060 | Cyclophilin, putative |  | N.D. |
| Lin125C8 | 2.35 | 1.2 ± 0.2 | 0.011 | 0 | 0 | b | LinJ.36.6770 | Histidine secretory acid phosphatase, putative | - | 1.4 ±0.4 |
|  |  |  |  |  |  |  | LinJ.36.6780 | Ubiquitin fusion degradation protein, putative | + | 2.3 ± 0.2 |
| Lin129D7 | 6.29 | 2.6 ± 0.1 | 0.001 | 0 | 0 | a | LinJ.36.4870 | Hypothetical protein, conserved |  | N.D. |
|  |  |  |  |  |  |  | LinJ.36.4880 | 60S acidic ribosomal protein, putative | + | 3.5 ± 0.1 |
|  |  |  |  |  |  |  | LinJ.36.4890 | Hypothetical protein, conserved |  | N.D. |
| Lin134A9 | 4.93 | 2.3 ± 0.3 | 0.007 | 0 | 0 | a | LinJ.36.2040 | Nucleoside transporter 1, putative | + | 2.4 ± 0.2 |
|  |  |  |  |  |  |  | LinJ.36.2050 | Mismatch repair protein MSH8, putative | + | 3.3 ± 0.1 |
| Lin134E11 | 3.90 | 2.0 ± 0.4 | 0.011 | 0 | 0 | a | LinJ.23.0060 | Cyclophilin, putative |  | N.D. |
| Lin135F1 | 2.18 | 1.1 ± 0.2 | 0.010 | 0 | 0 | b | LinJ.23.0020 | Hypothetical protein, conserved |  | N.D. |
|  |  |  |  |  |  |  | LinJ.23.0030 | Hypothetical protein, conserved |  | N.D. |
|  |  |  |  |  |  |  | LinJ.23.0040 | β-propeller protein, putative | + | 9.1 ± 0.6 |
|  |  |  |  |  |  |  | LinJ.23.0050 | Peroxidoxin (Tryparedoxin peroxidase) | **-** | 1.3 ± 0.1 |
| Lin132E1 | 2.20 | 1.1 ± 0.1 | 0.005 | 0 | 0 | c | LinJ.16.0450 | Fucose kinase, putative | + | 4.7 ± 0.4 |
| Lin139D8 | 4.68 | 2.2 ± 0.4 | 0.009 | 0 | 0 | b | LinJ.08.0010 | Adaptor complex protein (AP) 3 δ subunit 1, putative |  | N.D. |
| Lin148E8 | 2.39 | 1.2 ± 0.1 | 0.003 | 0 | 0 | b | LinJ.31.3180 | Fe/Zn transporter protein-like protein |  | N.D. |
|  |  |  |  |  |  |  | LinJ.31.3190 | Fe/Zn transporter protein-like protein |  | N.D. |
| Lin152C2 | 2.06 | 1.0 ± 0.3 | 0.021 | 0 | 0 | a | LinJ.18.0560 | Vacuolar ATP synthase subunit c, putative |  | N.D. |
| Lin154G9 | 4.65 | 2.2 ± 0.3 | 0.007 | 0 | 0 | b | LinJ.25.0080 | Poly(A)-binding protein 3, putative (PABP3) |  | N.D. |
| Lin154H12 | 4.70 | 2.2 ± 0.2 | 0.003 | 0 | 0 | b | LinJ.33.1770 | UDP-GlcNAc:PI a1-6 GlcNAc-transferase |  | N.D. |
| Lin158C1 | 2.98 | 1.6 ± 0.1 | 0.002 | 0 | 0 | b | LinJ.21.2140 | ATP synthase F1 subunit, γ protein, putative |  | N.D. |
| Lin165D12 | 2.23 | 1.1 ± 0.3 | 0.019 | 0 | 0 | a | LinJ.22.0470 | Hypothetical protein, conserved |  | N.D. |
|  |  |  |  |  |  |  | LinJ.22.0480 | Ubiquitin-conjugating enzyme-like protein | + | 12.8 ±1.2 |
| Lin166F2 | 9.47 | 3.2 ± 0.8 | 0.021 | 1e-177 | 0 | b | LinJ.21.0770 | ATP-binding cassette protein subfamily E, member 1, putative (ABCE1) |  | N.D. |
| Lin168C4 | 3.78 | 1.9 ± 0.4 | 0.017 | 0 | 0 | a | LinJ.36.3180 | Clathrin coat assembly protein-like protein | + | 2.0 ± 0.1 |
|  |  |  |  |  |  |  | LinJ.36.3190 | pre-mRNA branch site protein p14, putative | + | 43.2 ±1.5 |
|  |  |  |  |  |  |  | LinJ.36.3200 | Hypothetical protein, conserved |  | N.D. |
| Lin169E6 | 2.24 | 1.2 ± 0.1 | 0.001 | 0 | 0 | b | LinJ.32.0550 | Profilin, putative |  | N.D. |
| Lin172B9 | 4.16 | 2.1 ± 0.7 | 0.035 | 4e-88 | 6e-41 | b | LinJ.26.1680 | Sphingolipid δ-4 desaturase, putative | + | 2.1± 0.1 |
|  |  |  |  |  |  |  | LinJ.26.1690 | Cytochrome c oxidase, subunit V, coxV, putative | + | 2.1 ± 0.2 |
| Lin178A5 | 3.74 | 1.9 ± 0.1 | 0.001 | 0 | 0 | b | LinJ.36.1490 | Translation elongation factor 1β, putative |  | N.D. |
| Lin178D11 | 3.11 | 1.6 ± 0.2 | 0.006 | 2e-78 | 8e-99 | b | LinJ.27.2490 | Calpain-like cysteine peptidase |  | N.D. |
| Lin183H5 | 2.39 | 1.3 ± 0.4 | 0.032 | 0 | 0 | b | LinJ.34.1630 | Hypothetical protein, conserved |  | N.D. |
|  |  |  |  |  |  |  | LinJ.34.1640 | Kinesin, putative | + | 2.7 ± 0.2 |
| Lin187B12 | 3.83 | 1.9 ± 0.7 | 0.043 | 0 | 0 | a | LinJ.32.0110 | Mitochondrial carrier protein |  | N.D. |
| Lin187C7 | 5.90 | 2.6 ± 0.4 | 0.007 | 0 | 0 | b | LinJ.26.1680 | Sphingolipidδ-4 desaturase, putative | + | 2.1 ± 0.1 |
|  |  |  |  |  |  |  | LinJ.26.1690 | Cytochrome c oxidase, subunit V, coxV, putative | + | 2.1 ± 0.2 |
|  |  |  |  |  |  |  | LinJ.26.1700 | Hypothetical protein, conserved |  | N.D. |
| Lin187C10 | 14.86 | 3.9 ± 0.2 | 0.001 | 0 | 0 | b | LinJ.06.1320 | Pteridin transporter, putative |  | N.D. |
| Lin187H6 | 2.15 | 1.1 ± 0.2 | 0.009 | 0 | 0 | b | LinJ.34.1640 | Kinesin, putative |  | N.D. |
| Lin193F5 | 4.31 | 2.1 ± 0.2 | 0.004 | 0 | 0 | b | LinJ.36.2070 | Phosphomannomutase, putative (PMM) | - | 1.1 ± 0.2 |
|  |  |  |  |  |  |  | LinJ.36.2080 | Hypothetical protein, conserved |  | N.D. |
|  |  |  |  |  |  |  | LinJ.36.2090 | Serine/Threonine protein phosphatase 2B, catalytic subunit A2, putative | + | 4.0 ± 0.2 |
| Lin197D2 | 4.98 | 2.3 ± 0.5 | 0.015 | 0 | 0 | b | LinJ.07.0150 | Acyl-CoA dehydrogenase, mitochondrial precursor, putative | + | 2.1 ± 0.0 |
|  |  |  |  |  |  |  | LinJ.07.0160 | Hypothetical protein, conserved |  | N.D. |
|  |  |  |  |  |  |  | LinJ.07.0170 | 3-hydroxyacyl-ACP dehydratase, putative | + | 15.03 |
| Lin205D11 | 6.79 | 2.8 ± 0.3 | 0.005 | 0 | 0 | a | LinJ.29.2420 | Enoyl-CoA hidratase isomerase, putative | - | -1.2 ± 0.1 |
|  |  |  |  |  |  |  | LinJ.29.2430 | Mitogen activated protein kinase, putative | + | 9.6 ± 0.4 |
| Lin205F11 | 2.40 | 1.3 ± 0.1 | 0.002 | 0 | 0 | a | LinJ.12.0480 | Vacuolar ATP synthase subunit, putative | + | 14.0 ± 0.3 |
|  |  |  |  |  |  |  | LinJ.12.0490 | Glucose 6-phosphate isomerase (PGI) | - | -1.3 ± 0.3 |
| Lin206F10 | 2.07 | 1.0 ± 0.3 | 0.029 | 0 | 0 | b | LinJ.07.0410 | Protein kinase, putative |  | N.D. |
| Lin208F7 | 4.53 | 2.2 ± 0.4 | 0.013 | 0 | 0 | b | LinJ.30.3640 | Serine/Threonine protein kinase, putative |  | N.D. |
| Lin208H5 | 7.31 | 2.9 ± 0.5 | 0.012 | 0 | 0 | b | LinJ.28.2200 | DNA-directed RNA polymerase-like protein, putative |  | N.D. |
| Lin214H2 | 2.17 | 1.1 ± 0.1 | 0.006 | 0 | 0 | b | LinJ.06.0590 | 60S ribosomal protein L23a, putative |  | N.D. |
| Lin215C7 | 4.53 | 2.2 ± 0.2 | 0.002 | 1e-128 | 0 | b | LinJ.22.1340 | Serine/Threonine protein phosphatase, putative |  | N.D. |
| Lin217H5 | 2.02 | 1.0 ± 0.1 | 0.004 | 0 | 0 | b | LinJ.31.0860 | Lipase |  | N.D. |
|  |  |  |  |  |  |  | LinJ.31.0870 | Lipase precursor-like protein |  | N.D. |
| Lin254A4 | 2.42 | 1.3 ± 0.2 | 0.042 | 0 | 0 | b | LinJ.04.1250 | Actin (ACT) |  | N.D. |
| Lin269A6 | 3.67 | 1.9 ± 0.4 | 0.012 | 0 | 0 | a | LinJ.23.0060 | Cyclophilin, putative |  | N.D. |
|  |  |  |  |  |  |  | LinJ.23.0070 | Hypothetical protein, conserved |  | N.D. |
|  |  |  |  |  |  |  | LinJ.23.0080 | Agmatinase, putative |  | N.D. |
| Lin271B3 | 2.62 | 1.4 ± 0.2 | 0.009 | 0 | 0 | a | LinJ.31.0580 | Mevalonate kinase, putative |  | N.D. |
|  |  |  |  |  |  |  | LinJ.31.0590 | Amino acid transporter aATP11, putative | - | -1.1 ± 0.1 |
| Lin276A4 | 2.36 | 1.2 ± 0.4 | 0.030 | 0 | 0 | a | LinJ.30.0640 | Ribosome biogenesis regulatory protein (RRS1), putative | - | -1.4 ± 0.1 |
|  |  |  |  |  |  |  | LinJ.30.0650 | Histidyl-tRNA synthetase, putative | + | 2.0 ± 0.0 |
| Lin274G12 | 4.28 | 2.1 ± 0.2 | 0.003 | 0 | 0 | a | LinJ.23.0040 | β -propeller protein, putative | + | 5.0 ± 0.2 |
|  |  |  |  |  |  |  | LinJ.23.0050 | Peroxidoxin (Tryparedoxin peroxidase) | **-** | 1.3 ± 0.1 |
| Lin280D9 | 5.05 | 2.3 ± 0.9 | 0.043 | 0 | 0 | a | LinJ.36.3840 | Nudix hydrolase-like protein, putative | + | 7.8 ± 0.1 |
|  |  |  |  |  |  |  | LinJ.36.3850 | Hypothetical protein, conserved |  | N.D. |
| Lin285F5 | 5.26 | 2.4 ± 0.9 | 0.041 | 0 | 0 | a | LinJ.36.3610 | Glycosyl transferase-like protein | + | 7.2 ± 0.7 |
|  |  |  |  |  |  |  | LinJ.36.3620 | Hypothetical protein, conserved |  | N.D. |
| Lin287D1 | 2.31 | 1.2 ± 0.0 | 0.001 | 2e-56 | 0 | b | LinJ.26.1530 | Trifunctional enzyme α subunit, mitochondrial precursor-like protein |  | N.D. |
| Lin289A8 | 3.99 | 2.0 ± 0.7 | 0.035 | 0 | 0 | b | LinJ.07.0010 | Ubiquitin-activating enzyme E1, putative |  | N.D. |
| Lin289F11 | 4.89 | 2.3 ± 0.2 | 0.002 | 0 | 0 | b | LinJ.25.2260 | Hypothetical protein, conserved |  | N.D. |
|  |  |  |  |  |  |  | LinJ.25.2270 | Hypothetical protein, conserved |  | N.D. |
|  |  |  |  |  |  |  | LinJ.25.2280 | Vacuolar protein sorting-like protein, putative | + | 7.1 ± 0.3 |
| Lin298B12 | 3.06 | 1.6 ± 0.2 | 0.006 | 0 | 0 | a | LinJ.33.1410 | Glutamine aminotransferase, putative | + | 4.7 ± 0.6 |
|  |  |  |  |  |  |  | LinJ.33.1420 | QA-SNARE protein, putative | + | 22.2 ± 1.2 |
| Lin298H2 | 4.03 | 2.0 ± 0.1 | 0.001 | 0 | 0 | b | LinJ.22.1340 | Serine/Threonine protein phosphatase, putative |  | N.D. |
