# Supplementary material for: Influence of the Microenvironment in the Transcriptome of Leishmania infantum Promastigotes: Sand Fly versus Culture
Source: PLoS Negl Trop Dis. 2016 May 10;10(5):e0004693. doi: 10.1371/journal.pntd.0004693 (PMC4862625; doi:10.1371/journal.pntd.0004693)
Supplement: S5 Table — (DOC) [file pntd.0004693.s006.doc]

**S5 Table. Complete list of up-regulated genes of known function in Pro-Stat.** The following items are specified for each selected clone: fold change (F ≤ -2);, SD; Student’s t-test p-value; expect value in alignments (e-value); clone definition according to mapping outcomes a, b and c; Gene Id. retrieved from the database TriTrypDB; annotated functions in the *L. infantum* genome sequence; qRT-PCR outcomes. See more detailed information in the Methods section.

| ***Clone*** | ***F*** | ***-log_2_[F]± SD*** | ***p*** | ***e-value*** | | ***Def.*** | ***Id.*** | ***Annotated gene function*** | ***qRT-PCR*** | |
| --- | --- | --- | --- | --- | --- | --- | --- | --- | --- | --- |
|  |  |  |  | ***Fw*** | ***Rv*** |  |  |  |
| Lin16A10 | -2.25 | -1.2 ± 0.1 | 0.011 | 0 | 0 | b | LinJ.32.0460 | 40S ribosomal protein S2, putative | + | -2.6 ± 0.3 |
|  |  |  |  |  |  |  | LinJ.32.0470 | Prostaglandin F synthetase, putative | - | 1.1 ± 0.2 |
| Lin18A12 | -2.32 | -1.2 ± 0.5 | 0.046 | 0 | 0 | b | LinJ.33.2430 | UDP-glucose 4’-epimerase, putative |  | N.D. |
| Lin21B8 | -2.05 | -1.0 ± 0.3 | 0.037 | 0 | 2e-43 | b | LinJ.31.2420 | p-nitrophenylphosphatase, putative |  | N.D. |
| Lin24A5 | -2.31 | -1.2 ± 0.3 | 0.015 | 0 | 0 | b | LinJ.36.5240 | 40S ribosomal protein SA, putative |  | N.D. |
| Lin24C6 | -3.30 | -1.7 ± 0.1 | 0.003 | 0 | 0 | b | LinJ.36.5240 | 40S ribosomal protein SA, putative |  | N.D. |
| Lin25B7 | -2.73 | -1.4 ± 0.2 | 0.008 | 0 | 0 | b | LinJ.31.3390 | Sodium stibogluconate resistance protein, putative |  | N.D. |
| Lin27C2 | -2.78 | -1.5 ± 0.5 | 0.040 | 0 | 0 | b | LinJ.15.1070 | Glutamate dehydrogenase, putative (GDH) |  | N.D. |
| Lin30H4 | -4.73 | -2.2 ± 0.1 | 0.015 | 0 | 3e-107 | b | LinJ.27.2500 | Glycosomal phosphoenolpyruvate carboxykinase, putative |  | N.D. |
| Lin34B5 | -2.06 | -1.0 ± 0.1 | 0.004 | 2e-142 | 0 | b | LinJ.03.0190 | δ-1-pirroline-5-carboxylate dehydrogenase, putative |  | N.D. |
| Lin34G5 | -5.32 | -2.4 ± 0.9 | 0.043 | 0 | 0 | b | LinJ.06.0340 | Serine oligopeptidase B-like protein, Clan SC, familia S9A | - | 1.2 ± 0.2 |
|  |  |  |  |  |  |  | LinJ.06.0350 | NAD(P)-dependent steroid dehydrogenase-like protein | + | -2.0 ± 0.2 |
| Lin34E3 | -2.31 | -1.2 ± 0.4 | 0.040 | 9e-108 | 0 | b | LinJ.35.2830 | Transporter-like protein |  | N.D. |
| Lin34E10 | -2.64 | -1.4 ± 0.4 | 0.033 | 0 | 2e-154 | b | LinJ.35.2830 | Transporter-like protein |  | N.D. |
| Lin35H4 | -3.57 | -1.8 ± 0.4 | 0.013 | 0 | 0 | b | LinJ.34.3740 | Expression site-associated glycoprotein 5 (ESAG5) |  | N.D. |
| Lin50G2 | -2.04 | -1.0 ± 0.1 | 0.002 | 0 | 0 | b | LinJ.34.2660 | Amastin-like surface glycoprotein |  | N.D. |
| Lin50E2 | -3.56 | -1.8± 0.4 | 0.013 | 8e-22 | 0 | b | LinJ.07.0540 | Hypothetical protein, conserved |  | N.D. |
|  |  |  |  |  |  |  | LinJ.07.0550 | 60S ribosomal protein L7, putative | + | -2.1 ± 0.0 |
| Lin53D8 | -5.56 | -2.5 ± 0.2 | 0.002 | 0 | 0 | b | LinJ.30.3700 | Hypothetical protein, conserved |  | N.D. |
|  |  |  |  |  |  |  | LinJ.30.3710 | Ribosomal protein L15, putative | + | -2.0 ± 0.1 |
| Lin55D10 | -2.50 | -1.3 ± 0.1 | 0.004 | 0 | 0 | b | LinJ.18.1660 | γ-glutamylcysteine synthetase, putative (GSH1) |  | N.D. |
| Lin57H5 | -2.40 | -1.3 ± 0.2 | 0.009 | 0 | 0 | b | LinJ.08.0700 | Amastin-like protein |  | N.D. |
| Lin63A3 | -2.11 | -1.1 ± 0.4 | 0.036 | 0 | 0 | b | LinJ.34.3440 | 60S ribosomal protein L21, putative |  | N.D. |
| Lin63A8 | -2.21 | -1.1 ± 0.1 | 0.001 | 0 | 0 | a | LinJ.07.0060 | α-adaptin-like protein |  | N.D. |
| Lin79B10 | -3.08 | -1.6 ± 0.3 | 0.014 | 0 | 0 | b | LinJ.35.2580 | Hypothetical protein, unknown function |  | N.D. |
| Lin79F8 | -2.95 | -1.6 ± 0.1 | 0.003 | 8e-34 | 2e-34 | a | LinJ.24.1910 | Lysophospholipase, putative |  | N.D. |
| Lin80A1 | -2.43 | -1.3 ± 0.2 | 0.011 | 0 | 2e-65 | b | LinJ.36.6550 | Glucose transporter 2, putative |  | N.D. |
| Lin83D11 | -2.22 | -1.1 ± 0.4 | 0.046 | 0 | 0 | b | LinJ.08.0700/10 | Amastin-like protein |  | N.D. |
| Lin84B5 | -2.04 | -1.0 ± 0.3 | 0.032 | 0 | 0 | b | LinJ.34.3030 | α-keto acid decarboxylase, putative |  | N.D. |
| Lin91B12 | -2.70 | -1.4 ± 0.2 | 0.009 | 0 | 0 | b | LinJ.34.2660 | Amastin-like surface protein,putative |  | N.D. |
| Lin92F7 | -2.04 | -1.6 ± 0.1 | 0.002 | 0 | 0 | b | LinJ.35.0400 | 40S ribosomal protein S3a, putative |  | N.D. |
| Lin93C5 | -4.32 | -2.1 ± 0.4 | 0.009 | 0 | 1e-115 | b | LinJ.13.1410 | 60S ribosomal protein L44, putative |  | N.D. |
|  |  |  |  |  |  |  | LinJ.13.1420 | Pyrroline-5-carboxylate reductase (P5CR) |  | N.D. |
| Lin95F9 | -3.45 | -1.8 ± 0.4 | 0.016 | 0 | 0 | b | LinJ.28.2360 | Ribosomal protein S29, putative |  | N.D. |
| Lin101D5 | -3.61 | 1.8 ± 0.4 | 0.014 | 8e-31 | 0 | b | LinJ.27.2500 | Glycosomal phosphoenolpyruvate carboxykinase, putative |  | N.D. |
| Lin102E4 | -2.11 | -1.1 ± 0.4 | 0.038 | 1e-156 | 0 | b | LinJ.23.1200 | Hydrophilic acylated surface protein A (HASPA1) |  | N.D. |
| Lin104F8 | -2.71 | -1.4 ± 0.3 | 0.011 | 0 | 3e-129 | b | LinJ.24.1280 | Amastin-like surface protein-like protein |  | N.D. |
| Lin110A4 | -2.15 | -1.1 ± 0.3 | 0.028 | 0 | 0 | a | LinJ.24.1460 | Mismatch repair protein, putative |  | N.D. |
| Lin110F1 | -2.16 | -1.1 ± 0.3 | 0.013 | 0 | 2e-105 | b | LinJ.35.1850 | Protein kinase-like protein |  | N.D. |
| Lin110G8 | -2.22 | -1.1 ± 0.3 | 0.013 | 0 | 0 | b | LinJ.31.0480 | Calpain-like cysteine peptidase, Clan CA, family C2, putative |  | N.D. |
| Lin114E2 | -2.13 | -1.1 ± 0.2 | 0.011 | 0 | 0 | b | LinJ.36.7220 | Nuclear pore complex protein (NUP155)/nucleoporin, putative |  | N.D. |
| Lin123E6 | -3.38 | -1.8 ± 0.4 | 0.021 | 0 | 0 | b | LinJ.23.0980 | Actin-interacting protein |  | N.D. |
| Lin123D6 | -2.72 | -1.4 ± 0.1 | 0.001 | 0 | 0 | b | LinJ.34.2660 | Amastin-like surface protein, putative |  | N.D. |
| Lin125B1 | -2.56 | -1.4 ± 0.4 | 0.027 | 0 | 0 | b | LinJ.23.1060 | β-fructosidase/invertase/sucrose hydrolase-like |  | N.D. |
| Lin125H4 | -2.71 | -1.4 ± 0.2 | 0.008 | 0 | 0 | b | LinJ.36.1590 | Serine/Threonine protein kinase, putative |  | N.D. |
| Lin126D1 | -2.74 | -1.4 ± 0.2 | 0.007 | 0 | 0 | b | LinJ.35.2080 | Calcium motive P-type ATPase, putative |  | N.D. |
| Lin128D7 | -2.78 | -1.5 ± 0.5 | 0.030 | 0 | 0 | b | LinJ.21.2150 | 40S ribosomal protein S6, putative |  | N.D. |
| Lin134H1 | -2.64 | -1.4 ± 0.3 | 0.012 | 0 | 0 | b | LinJ.31.1310 | ATP-binding cassette protein subfamily c, member 6, putative (ABCC6) |  | N.D. |
| Lin166B10 | -2.25 | -1.2 ± 0.3 | 0.025 | 0 | 9e-37 | b | LinJ.31.1850 | Amino acid permease |  | N.D. |
| Lin166B5 | -2.27 | -1.2 ± 0.3 | 0.016 | 0 | 0 | b | LinJ.05.1210 | Surface antigen-like protein |  | N.D. |
| Lin172C4 | -3.88 | -2.0 ± 0.2 | 0.003 | 0 | 0 | b | LinJ.34.0820 | Serine/Threonine protein phosphatase 1, putative |  | N.D. |
|  |  |  |  |  |  |  | LinJ.34.0830 | Serine/Threonine protein phosphatase 1, putative |  | N.D. |
| Lin173E11 | -2.73 | -1.4 ± 0.4 | 0.023 | 5e-66 | 4e-36 | b | LinJ.36.2280 | COP-coated vesicle membrane RE-Golgi transport protein erv25 precursor |  | N.D. |
| Lin174F8 | -2.11 | -1.1 ± 0.1 | 0.001 | 0 | 0 | b | LinJ.08.0790 | Amastin-like protein |  | N.D. |
| Lin185C1 | -2.07 | -1.0 ± 0.4 | 0.048 | 0 | 0 | a | LinJ.11.0990 | Adaptin-related protein-like protein |  | N.D. |
| Lin188B12 | -3.68 | -1.9 ± 0.4 | 0.013 | 0 | 0 | b | LinJ.31.3400 | Sodium stibogluconate resistance protein |  | N.D. |
| Lin193E6 | -4.16 | -2.0 ± 0.3 | 0.007 | 0 | 0 | b | LinJ.23.1230 | Small hydrophilic endoplasmic reticulum-associated protein (SHERP) |  | N.D. |
| Lin194B9 | -4.38 | -2.1 ± 0.8 | 0.045 |  |  |  | LinJ.03.0600 | Mitochondrial protein MP99, putative |  | N.D. |
| Lin198D12 | -2.81 | -1.5 ± 0.2 | 0.007 | 0 | 0 | b | LinJ.24.2300 | 60S ribosomal protein L12, putative |  | N.D. |
| Lin202E7 | -2.42 | -1.3 ± 0.5 | 0.045 | 4e-153 | 0 | b | LinJ.31.2060 | Succinyl-diaminopimelate desuccinylase-like protein |  | N.D. |
| Lin205A5 | -2.38 | -1.2 ± 0.2 | 0.010 | 0 | 0 | a | LinJ.34.0950 | Ubiquitin-conjugating enzyme E2, putative |  | N.D. |
| Lin205E2 | -2.01 | -1.0 ± 0.2 | 0.012 | 2e-90 | 0 | b | LinJ.35.4060 | Protein kinase A catalytic subunit isoform 1 |  | N.D. |
| Lin231A5 | -2.06 | -1.0 ± 0.3 | 0.030 | 0 | 0 | b | LinJ.35.1310 | Ubiquitin-conjugating enzyme E2, putative |  | N.D. |
| Lin274G6 | -2.38 | -1.2 ± 0.3 | 0.020 | 0 | 0 | b | LinJ.08.0680/90 | Amastin-like protein |  | N.D. |
| Lin282B9 | -2.84 | -1.5 ± 0.5 | 0.040 | 0 | 0 | a | LinJ.23.0620 | Oxidoreductase-like protein |  | N.D. |
| Lin286D1 | -2.90 | -1.5 ± 0.2 | 0.011 | 0 | 5e-56 | b | LinJ.08.1320 | Amastin-like protein |  | N.D. |
| Lin287H2 | -2.17 | -1.1 ± 0.3 | 0.025 | 0 | 0 | b | LinJ.03.0190 | δ-1-pirroline-5-carboxylate dehydrogenase, putative |  | N.D. |
|  |  |  |  |  |  |  | LinJ.03.0200 | Protein kinase, putative |  | N.D. |
| Lin307F2 | -2.12 | -1.1 ± 0.2 | 0.007 | 0 | 0 | b | LinJ.11.0060 | Protein kinase, putative |  | N.D. |
| Lin307F7 | -2.06 | -1.0 ± 0.0 | 0.001 | 0 | 0 | b | LinJ.36.2860 | Hypothetical protein, conserved/Transportin 2-like |  | N.D. |
| Lin309F12 | -2.89 | -1.5 ± 0.3 | 0.013 | 5e-97 | 5e-60 | a | LinJ.17.0770 | POLO-like protein kinase, putative |  | N.D. |
