# Supplementary material for: Influence of the Microenvironment in the Transcriptome of Leishmania infantum Promastigotes: Sand Fly versus Culture
Source: PLoS Negl Trop Dis. 2016 May 10;10(5):e0004693. doi: 10.1371/journal.pntd.0004693 (PMC4862625; doi:10.1371/journal.pntd.0004693)
Supplement: S6 Table — (DOC) [file pntd.0004693.s007.doc]

**S6 Table. Hypothetical protein genes up-regulated in Pro-Pper/Pro-Stat.**

| *Clone* | *F* | *Log_2_F ± SD* | *p* | *e-value* | | *Def.* | *Id.* | *Annotated gene function* | *qRT-PCR* | |
| --- | --- | --- | --- | --- | --- | --- | --- | --- | --- | --- |
|  |  |  |  | *Fw* | *Rv* |  |  |  |  |  |
| Lin3A8 | 3.86 | 1.9 ± 0.3 | 0.009 | 5e-29 | 5e-174 | a | LinJ.31.2230 | Hypothetical protein, conserved |  | N.D. |
| Lin16B12 | 5.71 | 2.5 ± 0.1 | 0.000 | 0 | 0 | b | LinJ.25.1850 | 3-oxo-5-α-steroid 4-dehydrogenase, putative | - | 1.3 ± 0.1 |
|  |  |  |  |  |  |  | LinJ.25.1860 | Hypothetical protein, conserved |  | N.D. |
| Lin21H4 | 3.37 | 1.7 ± 0.3 | 0.009 | 0 | 0 | b | LinJ.31.1370 | Hypothetical protein, unknown function |  | N.D. |
| Lin22H5 | 4.82 | 2.3 ± 0.1 | 0.000 | 0 | 0 | b | LinJ.19.0180 | Hypothetical protein, conserved |  | N.D. |
|  |  |  |  |  |  |  | LinJ.19.0190 | ATP/ADP transporter protein 1, mitochondrial precursor, putative | - | -1.1 ± 0.0 |
| Lin26A1 | 4.43 | 2.1 ± 0.4 | 0.010 | 0 | 0 | b | LinJ.25.0090 | Hypothetical protein, conserved |  | N.D. |
|  |  |  |  |  |  |  | LinJ.25.0100 | Hypothetical protein, conserved |  | N.D. |
| Lin27H9 | 3.08 | 1.6 ± 0.3 | 0.012 | 0 | 0 | a | LinJ.17.0010 | Hypothetical protein, conserved |  | N.D. |
| Lin28F4 | 4.27 | 2.1 ± 0.3 | 0.007 | 0 | 0 | a | LinJ.33.0660 | Hypothetical protein, conserved |  | N.D. |
| Lin31A12 | 7.96 | 3.0 ± 0.5 | 0.010 | 0 | 0 | b | LinJ.19.1490 | Oxidoreductase-like protein | - | -1.1 ± 0.2 |
|  |  |  |  |  |  |  | LinJ.19.1500 | Hypothetical protein, conserved |  | N.D. |
| Lin35B6 | 3.96 | 2.0 ± 0.1 | 0.002 | 0 | 0 | b | LinJ.28.0740 | Hypothetical protein, conserved |  | N.D. |
| Lin43A9 | 4.04 | 2.0 ± 0.5 | 0.020 | 0 | 0 | a | LinJ.33.0660 | Hypothetical protein, conserved |  | N.D. |
|  |  |  |  |  |  |  | LinJ.33.0670 | Hypothetical protein, conserved |  | N.D. |
|  |  |  |  |  |  |  | LinJ.33.0680 | Hypothetical protein, conserved |  | N.D. |
| Lin43F3 | 8.84 | 3.1 ± 0.4 | 0.006 | 3e-98 | 5e-103 | a | LinJ.32.0500 | Hypothetical protein, conserved |  | N.D. |
| Lin49B2 | 4.33 | 2.1 ± 0.1 | 0.002 | 0 | 0 | b | LinJ.01.0290 | Hypothetical protein, conserved |  | N.D. |
|  |  |  |  |  |  |  | LinJ.01.0300 | Hypothetical protein, conserved |  | N.D. |
|  |  |  |  |  |  |  | LinJ.01.0310 | Acidocalcisome exopoylphosphatase, putative | - | -1.4 ± 0.2 |
| Lin50A12 | 2.04 | 1.0 ± 0.3 | 0.031 | 5e-171 | 0 | a | LinJ.32.1820 | Zinc finger protein 2, putative | - | 1.0 ± 0.1 |
|  |  |  |  |  |  |  | LinJ.32.1830 | Hypothetical protein, conserved |  | N.D. |
| Lin76B11 | 4.08 | 2.0 ± 0.1 | 0.004 | 0 | 0 | b | LinJ.33.1670 | Hypothetical protein, conserved |  |  |
| Lin100C10 | 3.60 | 1.8 ± 0.4 | 0.016 | 0 | 0 | a | LinJ.10.1350 | Hypothetical protein, conserved |  | N.D. |
|  |  |  |  |  |  |  | LinJ.10.1360 | Hypothetical protein, conserved |  | N.D. |
|  |  |  |  |  |  |  | LinJ.10.1370 | Hypothetical protein, conserved |  | N.D. |
| Lin100H5 | 4.00 | 2.0 ± 0.1 | 0.001 | 0 | 0 | b | LinJ.27.0490 | Hypothetical protein, conserved |  | N.D. |
| Lin105H10 | 7.40 | 2.9 ± 0.6 | 0.013 | 0 | 0 | b | LinJ.18.0570 | Hypothetical protein, conserved |  | N.D. |
| Lin106F1 | 4.94 | 2.3 ± 0.8 | 0.036 | 0 | 0 | b | LinJ.35.3970 | Hypothetical protein, conserved |  | N.D. |
| Lin107G9 | 11.65 | 3.5 ± 1.0 | 0.024 | 0 | 0 | b | LinJ.13.1430 | Hypothetical protein, conserved |  | N.D. |
|  |  |  |  |  |  |  | LinJ.13.1440 | Hypothetical protein, conserved |  | N.D. |
| Lin107H10 | 2.29 | 1.2 ± 0.2 | 0.010 | 8e-102 | 7e-133 | a | LinJ.24.1380 | Translation initiation factor IF2, putative | - | 1.2 ± 0.1 |
|  |  |  |  |  |  |  | LinJ.24.1390 | Hypothetical protein, conserved |  | N.D. |
| Lin113E12 | 3.72 | 1.9 ± 0.3 | 0.009 | 0 | 0 | a | LinJ.05.1050 | Hypothetical protein, unknown function |  | N.D. |
|  |  |  |  |  |  |  | LinJ.05.1060 | ATPase, putative | - | -1.1 ± 0.1 |
|  |  |  |  |  |  |  | LinJ.05.1070 | Hypothetical protein, conserved |  | N.D. |
| Lin128C11 | 4.38 | 2.1 ± 0.0 | 0.008 | 3e-166 | 7e-164 | b | LinJ.26.2000 | Hypothetical protein, conserved |  | N.D. |
| Lin129E3 | 4.45 | 2.1 ± 0.4 | 0.012 | 0 | 0 | a | LinJ.36.6610 | Hypothetical protein, conserved |  | N.D. |
|  |  |  |  |  |  |  | LinJ.36.6620 | Hypothetical protein, conserved |  | N.D. |
| Lin129E6 | 3.98 | 2.0 ± 0.4 | 0.011 | 0 | 0 | a | LinJ.22.1460 | Hypothetical protein, conserved |  | N.D. |
|  |  |  |  |  |  |  | LinJ.22.1470 | Hypothetical protein, conserved |  | N.D. |
| Lin131F11 | 4.82 | 2.3 ± 0.4 | 0.008 | 0 | 0 | a | LinJ.34.0040 | Hypothetical protein, conserved |  | N.D. |
|  |  |  |  |  |  |  | LinJ.34.0050 | Hypothetical protein, conserved |  | N.D. |
| Lin133H10 | 2.32 | 1.2 ± 0.4 | 0.036 | 0 | 0 | b | LinJ.36.1420 | Transitional endoplasmic reticulum ATPase/valosine, putative | - | 1.3 ± 0.1 |
|  |  |  |  |  |  |  | LinJ.36.1430 | Hypothetical protein, conserved |  | N.D. |
| Lin134F9 | 3.69 | 1.9 ± 0.2 | 0.003 | 0 | 5e-26 | b | LinJ.32.3150 | Hypothetical protein, unknown function |  | N.D. |
| Lin135A8 | 3.12 | 1.6 ± 0.3 | 0.014 | 0 | 0 | a | LinJ.35.4270 | Hypothetical protein, conserved |  | N.D. |
| Lin135B3 | 3.99 | 2.0 ± 0.4 | 0.013 | 0 | 0 | a | LinJ.30,0750 | Hypothetical protein, conserved |  | N.D. |
|  |  |  |  |  |  |  | LinJ.30.0760 | Co-chaperone GrpE, putative | - | -1.2 ± 0.0 |
| Lin136C5 | 3.10 | 1.6 ± 0.4 | 0.024 | 0 | 0 | b | LinJ.35.5030 | Hypothetical protein, conserved |  | N.D. |
| Lin136D8 | 3.06 | 1.6 ± 0.6 | 0.041 | 0 | 0 | a | LinJ.06.0650 | Hypothetical protein, conserved |  | N.D. |
|  |  |  |  |  |  |  | LinJ.06.0660 | Hypothetical protein, conserved |  | N.D. |
|  |  |  |  |  |  |  | LinJ.06.0670 | Hypothetical protein, conserved |  | N.D. |
| Lin138A8 | 5.09 | 2.3 ± 0.4 | 0.012 | 0 | 0 | a | LinJ.29.0980 | Hypothetical protein, conserved |  | N.D. |
| Lin138H11 | 3.11 | 1.6 ± 0.5 | 0.034 | 0 | 0 | b | LinJ.35.1380 | Hypothetical protein, unknown function |  | N.D. |
| Lin139A5 | 5.83 | 2.5 ± 0.6 | 0.017 | 0 | 0 | b | LinJ.11.0570 | Hypothetical protein, conserved |  | N.D. |
|  |  |  |  |  |  |  | LinJ.11.0580 | Tetratricopeptide repeat domain protein, putative | - | 1.5 ± 0.1 |
| Lin139E9 | 4.14 | 2.0 ± 0.2 | 0.003 | 0 | 0 | a | LinJ.27.1190 | Hypothetical protein, conserved |  | N.D. |
|  |  |  |  |  |  |  | LinJ.27.1200 | Hypothetical protein, conserved |  | N.D. |
| Lin149D5 | 3.03 | 2.0 ± 01 | 0.00 | 0 | 0 | b | LinJ.32.2530 | Hypothetical protein, conserved |  | N.D. |
|  |  |  |  |  |  |  | LinJ.32.2540 | Hypothetical protein, conserved |  | N.D. |
| Lin150C1 | 3.86 | 1.9 ± 0.6 | 0.032 | 0 | 0 | b | LinJ.33.1900 | Hypothetical protein, conserved |  | N.D. |
|  |  |  |  |  |  |  | LinJ.33.1910 | Hypothetical protein, conserved |  | N.D. |
| Lin155C10 | 11.61 | 3.5 ± 0.9 | 0.021 | 0 | 0 | a | LinJ.22.1330 | Hypothetical protein, conserved |  | N.D. |
| Lin155D4 | 3.93 | 2.0 ± 0.5 | 0.023 | 1e-23 | 0 | a | LinJ.32.2300 | Hypothetical protein, conserved |  | N.D. |
| Lin159D10 | 3.42 | 1.8 ± 0.2 | 0.003 | 0 | 0 | b | LinJ.35.2920 | Hypothetical protein, conserved |  | N.D. |
|  |  |  |  |  |  |  | LinJ.35.2930 | Hypothetical protein, conserved |  | N.D. |
| Lin161G4 | 4.40 | 2.1 ± 0.4 | 0.014 | 0 | 9e-151 | b | LinJ.30.2310 | Hypothetical protein, conserved |  | N.D. |
|  |  |  |  |  |  |  | LinJ.30.2320 | Hypothetical protein, conserved |  | N.D. |
|  |  |  |  |  |  |  | LinJ.30.2330 | Hypothetical protein, conserved |  | N.D. |
| Lin162H6 | 4.56 | 2.2 ± 0.5 | 0.017 | 0 | 0 | a | LinJ.31.1330 | Hypothetical protein, conserved |  | N.D. |
| Lin169B8 | 3.13 | 1.6 ± 0.1 | 0.001 | 0 | 3e-21 | b | LinJ.24.2320 | Multiple transmembrane domain hypothetical protein | - | 1.3 ± 0.2 |
|  |  |  |  |  |  |  | LinJ.24.2330 | Hypothetical protein, conserved |  | N.D. |
| Lin170C11 | 2.84 | 1.5 ± 0.2 | 0.008 | 0 | 0 | a | LinJ.30.0810 | Hypothetical protein, conserved |  | N.D. |
|  |  |  |  |  |  |  | LinJ.30.0820 | Hypothetical protein, conserved |  | N.D. |
|  |  |  |  |  |  |  | LinJ.30.0830 | Oligo(U)-binding mitochondrial protein TBRGG1, putative |  | N.D. |
| Lin172D9 | 2.52 | 1.3 ± 0.2 | 0.006 | 0 | 6e-143 | b | LinJ.22.0600 | NADH-dependent cytochrome b5 reductase, putative | - | -1.1 ± 0.1 |
|  |  |  |  |  |  |  | LinJ.22.0610 | Hypothetical protein, conserved |  | N.D. |
| Lin177A2 | 3.70 | 1.9 ± 0.5 | 0.026 | 0 | 0 | a | LinJ.35.4680 | Hypothetical protein, conserved |  | N.D. |
|  |  |  |  |  |  |  | LinJ.35.4690 | Hypothetical protein, conserved |  | N.D. |
| Lin179B8 | 4.42 | 2.1 ± 0.6 | 0.025 | 0 | 0 | b | LinJ.32.0880 | Hypothetical protein, unknown function |  | N.D. |
| Lin183B6 | 3.98 | 2.0 ± 0.0 | 0.000 | 0 | 0 | a | LinJ.23.0100 | Hypothetical protein, conserved |  | N.D. |
|  |  |  |  |  |  |  | LinJ.23.0110 | Hypothetical protein, conserved |  | N.D. |
| Lin200D7 | 2.28 | 1.2 ± 0.4 | 0.037 | 0 | 3e-58 | b | LinJ.35.1580 | Metacaspase, putative | - | 1.1 ± 0.1 |
|  |  |  |  |  |  |  | LinJ.35.1590 | Hypothetical protein, conserved |  | N.D. |
| Lin203B5 | 11.39 | 3.5 ± 0.4 | 0.004 | 0 | 0 | b | LinJ.32.0880 | Hypothetical protein, unknown function |  | N.D. |
|  |  |  |  |  |  |  | LinJ.32.0890 | Hypothetical protein, conserved |  | N.D. |
| Lin204D8 | 3.95 | 2.0 ± 0.7 | 0.039 | 0 | 0 | a | LinJ.33.0650 | Hypothetical protein, conserved |  | N.D. |
|  |  |  |  |  |  |  | LinJ.33.0660 | Hypothetical protein, conserved |  | N.D. |
|  |  |  |  |  |  |  | LinJ.33.0670 | Hypothetical protein, conserved |  | N.D. |
| Lin228B9 | 7.22 | 2.8 ± 0.1 | 0.001 | 0 | 0 | b | LinJ.06.1360 | Hypothetical protein, conserved |  | N.D. |
| Lin228D4 | 3.28 | 1.7 ± 0.1 | 0.002 | 0 | 0 | a | LinJ.19.0080 | Hypothetical protein, conserved |  | N.D. |
|  |  |  |  |  |  |  | LinJ.19.0090 | Fibrillarin, putative | - | 1.0 ± 0.1 |
| Lin231E4 | 4.07 | 2.0 ± 0.7 | 0.034 | 0 | 0 | a | LinJ.06.1360 | Hypothetical protein, conserved |  | N.D. |
| Lin239C5 | 3.04 | 1.6 ± 0.2 | 0.002 | 0 | 0 | a | LinJ.28.2210 | Hypothetical protein, conserved |  | N.D. |
|  |  |  |  |  |  |  | LinJ.28.2220 | DEAD mitochondrial protein, putative | - | -1.1 ± 0.0 |
| Lin262A6 | 6.19 | 2.6 ± 1.0 | 0.048 | 0 | 0 | b | LinJ.36.4810 | Hypothetical protein, conserved |  | N.D. |
| Lin267B9 | 3.08 | 1.6 ± 0.2 | 0.006 | 0 | 0 | b | LinJ.36.0580 | Hypothetical protein, conserved |  | N.D. |
|  |  |  |  |  |  |  | LinJ.36.0590 | Ubiquitin-like protein, putative | - | -1.3 ± 0.0 |
|  |  |  |  |  |  |  | LinJ.36.0600 | Protein kinase cdc2-related protein, putative | - | -1.1 ± 0.1 |
| Lin267D1 | 7.55 | 2.9 ± 0.7 | 0.021 | 0 | 0 | a | LinJ.19.1500 | Hypothetical protein, conserved |  | N.D. |
| Lin268E7 | 6.59 | 2.7 ± 0.5 | 0.010 | 0 | 0 | b | LinJ.26.2300 | Hypothetical protein, conserved |  | N.D. |
| Lin291A7 | 2.14 | 1.1 ± 0.4 | 0.039 | 0 | 0 | b | LinJ.36.7300 | Hypothetical protein, conserved/elks δ-like protein | - | -1.1 ± 0.0 |
|  |  |  |  |  |  |  | LinJ.36.7310 | Hypothetical protein, conserved |  | N.D. |
| Lin292E2 | 4.85 | 2.3 ± 0.3 | 0.006 | 0 | 0 | a | LinJ.36.6610 | Hypothetical protein, conserved |  | N.D. |
| Lin299E11 | 3.63 | 1.9 ± 0.7 | 0.041 | 0 | 0 | a | LinJ.36.4870 | Hypothetical protein, conserved |  | N.D. |
