# Supplementary material for: Influence of the Microenvironment in the Transcriptome of Leishmania infantum Promastigotes: Sand Fly versus Culture
Source: PLoS Negl Trop Dis. 2016 May 10;10(5):e0004693. doi: 10.1371/journal.pntd.0004693 (PMC4862625; doi:10.1371/journal.pntd.0004693)
Supplement: S7 Table — (DOC) [file pntd.0004693.s008.doc]

**S7 Table. Hypothetical protein genes down-regulated in Pro-Pper/Pro-Stat.**

| *Clone* | *F* | *Log_2_F ± SD* | *p* | *e-value* | | *Def.* | *Id.* | *Annotated gene function* | *qRT-PCR* | |
| --- | --- | --- | --- | --- | --- | --- | --- | --- | --- | --- |
|  |  |  |  | *Fw* | *Rv* |  |  |  |  |  |
| Lin3C7 | -2.18 | -1.1 ± 0.1 | 0.006 | 0 | 0 | b | LinJ.29.0310 | Hypothetical protein, conserved |  | N.D. |
| Lin4F1 | -4.52 | -2.2 ± 0.2 | 0.003 | 0 | 0 | b | LinJ.18.0660 | Hypothetical protein, conserved |  | N.D. |
| Lin9H9 | -2.22 | -1.1 ± 0.0 | 0.001 | 0 | 0 | b | LinJ.25.1440 | Hypothetical protein, conserved |  | N.D. |
| Lin20G7 | -2.60 | -1.4 ± 0.2 | 0.005 | 0 | 0 | a | LinJ.29.2870 | Hypothetical protein, conserved |  | N.D. |
| Lin21A12 | -2.31 | -1.2 ± 0.3 | 0.025 | 0 | 0 | b | LinJ.30.2660 | Hypothetical protein, conserved |  | N.D. |
|  |  |  |  |  |  |  | LinJ.30.2670 | Hypothetical protein, conserved |  | N.D. |
| Lin22A11 | -2.51 | -1.3 ± 0.3 | 0.016 | 0 | 3-172 | b | LinJ.33.0400 | Hypothetical protein, conserved |  | N.D. |
| Lin22D3 | -2.37 | -1.2 ± 0.1 | 0.001 | 0 | 0 | b | LinJ.06.1250 | Hypothetical protein, unknown function |  | N.D. |
|  |  |  |  |  |  |  | LinJ.06.1260 | Hypothetical protein, unknown function |  | N.D. |
| Lin26G6 | -2.89 | -1.5 ± 0.2 | 0.004 | 7e-93 | 1e-97 | a | LinJ.19.0880 | Hypothetical protein, conserved |  | N.D. |
| Lin30C11 | -4.2 | -2.1 ± 0.3 | 0.006 | 0 | 0 | a | LinJ.30.2400 | Hypothetical protein, conserved |  | N.D. |
| Lin31A8 | - 2.04 | -1.0 ± 0.2 | 0.014 | 0 | 0 | b | LinJ.19.1170 | Hypothetical protein, conserved |  | N.D. |
|  |  |  |  |  |  |  | LinJ.19.1180 | Hypothetical protein, conserved |  | N.D. |
| Lin31F6 | -2.11 | -1.1 ± 0.2 | 0.014 | 3e-126 | 0 | b | LinJ.35.0180 | Hypothetical protein, conserved |  | N.D. |
|  |  |  |  |  |  |  | LinJ.35.0190 | Hypothetical protein, conserved |  | N.D. |
| Lin32H7 | -3.37 | -1.7 ± 0.1 | 0.001 | 2e-151 | 6e-112 | a | LinJ.31.1380 | Hypothetical protein, conserved |  | N.D. |
| Lin34F12 | -2.57 | -1.4 ± 0.2 | 0.007 | 0 | 0 | a | LinJ.03.0290 | Hypothetical protein, conserved |  | N.D. |
| Lin37B5 | -2.75 | -1.5 ± 0.1 | 0.002 | 0 | 0 | a | LinJ.33.0570 | Hypothetical protein, conserved |  | N.D. |
|  |  |  |  |  |  |  | LinJ.33.0580 | Hypothetical protein, conserved |  | N.D. |
| Lin42H2 | -2.16 | -1.1 ± 0.2 | 0.007 | 0 | 0 | a | LinJ.35.3600 | Hypothetical protein, conserved |  | N.D. |
| Lin46B3 | -3.16 | -1.7 ± 0.4 | 0.017 | 0 | 0 | a | LinJ.32.3750 | Hypothetical protein, conserved |  | N.D. |
|  |  |  |  |  |  |  | LinJ.32.3760 | Hypothetical protein, conserved |  | N.D. |
|  |  |  |  |  |  |  | LinJ.32.3770 | Hypothetical protein, conserved |  | N.D. |
|  |  |  |  |  |  |  | LinJ.32.3780 | Hypothetical protein, conserved |  | N.D. |
| Lin50H6 | -8.73 | -3.1 ± 0.1 | 0.001 | 0 | 0 | b | LinJ.18.0480 | Uracyl DNA glycosylase, putative | - | -1.0 ± 0.0 |
|  |  |  |  |  |  |  | LinJ.18.0490 | Hypothetical protein, conserved |  | N.D. |
| Lin62E10 | -2.80 | -1.5 ± 0.5 | 0.003 | 0 | 0 | b | LinJ.35.3440 | Hypothetical protein, conserved |  | N.D. |
|  |  |  |  |  |  |  | LinJ.35.3450 | Hypothetical protein, conserved |  | N.D. |
| Lin68A12 | -2.08 | -1.0 ± 0.4 | 0.038 | 0 | 0 | a | LinJ.09.0320 | Hypothetical protein, conserved |  | N.D. |
| Lin69C11 | -2.41 | -1.3 ± 0.4 | 0.038 | 0 | 0 | b | LinJ.15.0210 | Hypothetical protein, conserved |  | N.D. |
| Lin74B8 | -2.41 | -1.3 ± 0.1 | 0.001 | 0 | 5e-134 | b | LinJ.17.0990 | Hypothetical protein, conserved |  | N.D. |
|  |  |  |  |  |  |  | LinJ.17.1000 | Hypothetical protein, conserved |  | N.D. |
| Lin74G10 | -2.84 | -1.5 ± 0.4 | 0.025 | 0 | 0 | b | LinJ.14.1410 | Hypothetical protein, conserved |  | N.D. |
| Lin79B10 | -3.08 | -1.6 ± 0.3 | 0.014 | 0 | 0 | b | LinJ.35.2580 | Hypothetical protein, unknown function |  | N.D. |
| Lin78C9 | -2.51 | -1.3 ± 0.3 | 0.016 | 0 | 0 | b | LinJ.32.0340 | Hypothetical protein, unknown function |  | N.D. |
| Lin90H3 | -2.58 | -1.4 ± 0.1 | 0.002 | 0 | 0 | a | LinJ.19.1540 | Hypothetical protein, conserved |  | N.D. |
| Lin92C5 | -2.49 | -1.3 ± 0.4 | 0.024 | 0 | 0 | b | LinJ.23.0910 | Hypothetical protein, conserved |  | N.D. |
| Lin95E6 | -2.79 | -1.5 ± 0.2 | 0.008 | 0 | 2e-179 | b | LinJ.16.1080 | Hypothetical protein, conserved |  | N.D. |
| Lin99A12 | -2.03 | -1.0 ± 0.1 | 0.002 | 0 | 0 | b | LinJ.33.0160 | Hypothetical protein, conserved |  | N.D. |
|  |  |  |  |  |  |  | LinJ.33.0170 | Hypothetical protein, unknown function |  | N.D. |
| Lin99F11 | -2.35 | -1.2 ± 0.4 | 0.032 | 0 | 0 | a | LinJ.30.3250 | Hypothetical protein, conserved |  | N.D. |
|  |  |  |  |  |  |  | LinJ.30.3260 | Hypothetical protein, conserved |  | N.D. |
|  |  |  |  |  |  |  | LinJ.30.3270 | Hypothetical protein, conserved |  | N.D. |
| Lin104C12 | -2.30 | -1.2 ± 0.0 | 0.017 | 0 | 0 | b | LinJ.18.0390 | Hypothetical protein, conserved |  | N.D. |
| Lin104D12 | -2.32 | -1.2 ± 0.6 | 0.044 | 0 | 0 | a | LinJ.32.1840 | Hypothetical protein, conserved |  | N.D. |
|  |  |  |  |  |  |  | LinJ.32.1850 | Hypothetical protein, conserved |  | N.D. |
|  |  |  |  |  |  |  | LinJ.32.1860 | Hypothetical protein, conserved |  | N.D. |
| Lin105G12 | -2.44 | -1.3 ± 0.5 | 0.040 | 1e-20 | 1e-29 | b | LinJ.29.1510 | Hypothetical protein, conserved |  | N.D. |
| Lin106B12 | -3.61 | -1.8 ± 0.5 | 0.021 | 0 | 0 | b | LinJ.29.2330 | Hypothetical protein, unknown function |  | N.D. |
| Lin109B11 | -2.66 | -1.4 ± 0.2 | 0.005 | 0 | 0 | b | LinJ.22.1070 | Hypothetical protein, conserved |  | N.D. |
| Lin111D1 | -2.32 | -1.2 ± 0.3 | 0.016 | 0 | 0 | a | LinJ.31.2630 | Hypothetical protein, conserved |  | N.D. |
| Lin111G5 | -2.27 | -1.2 ± 0.1 | 0.006 | 0 | 0 | b | LinJ.31.2630 | Hypothetical protein, conserved |  | N.D. |
| Lin120D4 | -2.10 | -1.1 ± 0.3 | 0.019 | 0 | 0 | b | LinJ.36.2960 | Hypothetical protein, conserved |  | N.D. |
| Lin120G5 | -2.69 | -1.4 ± 0.3 | 0.014 | 0 | 0 | a | LinJ.31.0490 | Hypothetical protein, conserved |  | N.D. |
| Lin121B2 | - 3.58 | -1.8 ± 0.1 | 0.001 | 0 | 4e-153 | b | LinJ.30.1770 | Hypothetical protein, conserved |  | N.D. |
| Lin123G2 | -2.41 | -1.3 ± 0.3 | 0.002 | 0 | 0 | b | LinJ.24.1940 | Hypothetical protein, conserved |  | N.D. |
| Lin124D2 | -2.73 | -1.4 ± 0.3 | 0.014 | 0 | 0 | a | LinJ.09.105 | Hypothetical protein, conserved |  | N.D. |
| Lin125A5 | -2.77 | -1.5 ± 0.5 | 0.032 | 0 | 0 | b | LinJ.32.2410 | Hypothetical protein, conserved |  | N.D. |
| Lin126F11 | -2.96 | -1.6 ± 0.2 | 0.006 | 0 | 0 | a | LinJ.09.1260 | Hypothetical protein, conserved |  | N.D. |
|  |  |  |  |  |  |  | LinJ.09.1270 | Hypothetical protein, unknown function |  | N.D. |
| Lin130H3 | -2.02 | -1.0 ± 0.3 | 0.021 | 0 | 0 | a | LinJ.27.1550 | Hypothetical protein, conserved |  | N.D. |
| Lin131G3 | -2.32 | -1.2 ± 0.1 | 0.005 | 8e-179 | 0 | b | LinJ.35.3770 | Hypothetical protein, conserved |  | N.D. |
| Lin131H9 | -2.00 | -1.0 ± 0.2 | 0.018 | 0 | 0 | b | LinJ.04.0740 | Hypothetical protein, conserved |  | N.D. |
| Lin133B9 | -2.11 | -1.1 ± 0.1 | 0.004 | 0 | 4e-57 | b | LinJ.31.1670 | Hypothetical protein, conserved |  | N.D. |
|  |  |  |  |  |  |  | LinJ.31.1680 | Hypothetical protein, conserved |  | N.D. |
| Lin137A6 | -2.49 | -1.3 ± 0.2 | 0.005 | 0 | 0 | a | LinJ.12.0360 | Hypothetical protein |  | N.D. |
|  |  |  |  |  |  |  | LinJ.12.0370 | Hypothetical protein, conserved |  | N.D. |
| Lin142A8 | -2.02 | -1.0 ± 0.2 | 0.014 | 5e-177 | 0 | b | LinJ.21.0850 | Hypothetical protein, conserved |  | N.D. |
| Lin143A11 | -2.63 | -1.0 ± 0.4 | 0.022 | 0 | 0 | b | LinJ.13.0200 | Hypothetical protein, conserved |  | N.D. |
| Lin150A5 | -5.35 | -2.4 ± 0.5 | 0.014 | 0 | 0 | b | LinJ.30.2310 | Hypothetical protein, conserved |  | N.D. |
|  |  |  |  |  |  |  | LinJ.30.2320 | Hypothetical protein, conserved |  | N.D. |
|  |  |  |  |  |  |  | LinJ.30.2330 | Hypothetical protein, conserved |  | N.D. |
| Lin155F1 | -2.5 | -1.3 ± 0.2 | 0.010 | 0 | 0 | b | LinJ.31.1100 | Hypothetical protein, conserved |  | N.D. |
| Lin157A11 | -2.03 | -1.0 ± 0.1 | 0.002 | 0 | 0 | b | LinJ.35.3250 | Hypothetical protein, conserved |  | N.D. |
| Lin158D6 | -3.12 | -1.6 ± 0.1 | 0.003 | 0 | 0 | b | LinJ.15.0060 | Hypothetical protein, conserved |  | N.D. |
| Lin159B5 | -3.35 | -1.7 ± 0.2 | 0.004 | 0 | 0 | b | LinJ.34.1990 | Hypothetical protein, conserved |  | N.D. |
| Lin161G12 | -3.06 | -1.6 ± 0.5 | 0.031 | 0 | 0 | b | LinJ.30.2310 | Hypothetical protein, conserved |  | N.D. |
| Lin165B10 | -2.82 | -1.5 ± 0.4 | 0.019 | 0 | 0 | b | LinJ.30.1620 | Hypothetical protein, conserved |  | N.D. |
| Lin166F9 | -2.52 | -1.3 ± 0.3 | 0.016 | 4e-116 | 3e-70 | b | LinJ.30.2310 | Hypothetical protein, conserved |  | N.D. |
|  |  |  |  |  |  |  | LinJ.30.2320 | Hypothetical protein, conserved |  | N.D. |
|  |  |  |  |  |  |  | LinJ.30.2330 | Hypothetical protein, conserved |  | N.D. |
| Lin169B11 | -2.93 | -1.5 ± 0.4 | 0.025 | 0 | 4e-162 | b | LinJ.06.1130 | Hypothetical protein, conserved |  | N.D. |
|  |  |  |  |  |  |  | LinJ.06.1140 | Hypothetical protein, conserved |  | N.D. |
| Lin173B8 | -2.13 | -1.1 ± 0.2 | 0.016 | 0 | 0 | b | LinJ.16.1080 | Hypothetical protein, conserved |  | N.D. |
| Lin174B9 | -3.92 | -2.0 ± 0.2 | 0.003 | 9e-179 | 2e-31 | b | LinJ.30.2310 | Hypothetical protein, conserved |  | N.D. |
|  |  |  |  |  |  |  | LinJ.30.2320 | Hypothetical protein, conserved |  | N.D. |
|  |  |  |  |  |  |  | LinJ.30.2330 | Hypothetical protein, conserved |  | N.D. |
| Lin174H9 | -3.07 | -1.6 ± 0.3 | 0.011 | 0 | 0 | a | LinJ.32.1580 | Hypothetical protein |  | N.D. |
| Lin181G3 | -2.68 | -1.4 ± 0.2 | 0.005 | 0 | 0 | b | LinJ.13.0600 | Hypothetical protein, conserved |  | N.D. |
| Lin181H11 | -6.70 | -2.7 ± 0.6 | 0.015 | 0 | 0 | a | LinJ.18.0720 | Hypothetical protein, conserved |  | N.D. |
| Lin185D3 | -2.23 | -1.2 ± 0.1 | 0.004 | 0 | 0 | b | LinJ.22.1070 | Hypothetical protein, conserved |  | N.D. |
| Lin188A7 | -2.15 | -1.1 ± 0.2 | 0.010 | 0 | 0 | b | LinJ.23.1360 | Hypothetical protein, conserved |  | N.D. |
| Lin188C7 | -2.16 | -1.1 ± 0.2 | 0.013 | 0 | 0 | a | LinJ.33.0430 | Hypothetical protein, conserved |  | N.D. |
| Lin206E8 | -3.16 | -1.7± 0.7 | 0.048 | 0 | 0 | b | LinJ.36.0900 | Hypothetical protein, conserved |  | N.D. |
| Lin207B7 | -2.65 | -1.4 ± 0.4 | 0.026 | 0 | 0 | b | LinJ.23.0650 | Hypothetical protein, conserved |  | N.D. |
|  |  |  |  |  |  |  | LinJ.23.0660 | Hypothetical protein, conserved |  | N.D. |
| Lin209A6 | -2.68 | -1.4 ± 0.2 | 0.005 | 0 | 0 | b | LinJ.22.1070 | Hypothetical protein, conserved |  | N.D. |
| Lin209F9 | -2.08 | -1.0 ± 0.3 | 0.035 | 0 | 2e-90 | b | LinJ.36.4970 | Hypothetical protein, conserved |  | N.D. |
| Lin210F2 | -4.19 | -2.1 ± 0.4 | 0.017 | 0 | 0 | b | LinJ.32.1100 | Hypothetical protein, conserved |  | N.D. |
| Lin226F8 | -2.26 | -1.2 ± 0.1 | 0.005 | 2e-142 | 0 | b | LinJ.26.0710 | Hypothetical protein, conserved |  | N.D. |
| Lin251B6 | -2.77 | -1.5 ± 0.2 | 0.009 | 0 | 0 | b | LinJ.31.1470 | Hypothetical protein, unknown function |  | N.D. |
