# Supplementary material for: Influence of the Microenvironment in the Transcriptome of Leishmania infantum Promastigotes: Sand Fly versus Culture
Source: PLoS Negl Trop Dis. 2016 May 10;10(5):e0004693. doi: 10.1371/journal.pntd.0004693 (PMC4862625; doi:10.1371/journal.pntd.0004693)
Supplement: S8 Table — (DOC) [file pntd.0004693.s009.doc]

**S8 Table. Type c and qPCR-undetermined clones.**

| *Clone* | *F* | *Log_2_F ± SD* | *p* | *e-value* | | *Def.* | *Id.* | *Annotated gene function* |
| --- | --- | --- | --- | --- | --- | --- | --- | --- |
|  |  |  |  | *Fw* | *Rv* |  |  |  |
| Lin23C8 | 3.25 | 1.7 ± 0.4 | 0.017 | 0 | 0 | b | LinJ.08.0300 | Vacuolar-type proton-translocating protein |
|  |  |  |  |  |  |  | LinJ.08.0040 | Hypothetical protein, conserved |
|  |  |  |  |  |  |  | LinJ.08.0050 | Hypothetical protein, unknown function |
| Lin104C1 | 3.48 | 1.8 ± 0.4 | 0.015 | 0 | 0 | a | LinJ.33.2920 | Hypothetical protein, conserved |
|  |  |  |  |  |  |  | LinJ.33.2930 | GTP-dependent elongation factor, family Tu, putative |
| Lin138C1 | 4.07 | 2.0 ± 0.2 | 0.005 | 0 | 0 | a | LinJ.24.1360 | Hypothetical protein, conserved |
|  |  |  |  |  |  |  | LinJ.24.1370 | Hypothetical protein, conserved |
|  |  |  |  |  |  |  | LinJ.24.1380 | Translation initiation factor IF2, putative |
| Lin155A2 | 2.82 | 1.5 ± 0.2 | 0.007 | 0 | 0 | b | LinJ.28.2280 | Dynein light chain LC6, flagellar outer arm, putative |
|  |  |  |  |  |  |  | LinJ.28.2890 | A/G-specific adenine glycosylase, putative |
| Lin173B9 | 2.21 | 1.1 ±0.3 | 0.028 | 0 | - | c | LinJ.30.2350 | Zinc finger protein, putative |
| Lin175B5 | 5.03 | 2.3 ± 0.6 | 0.024 | 0 | 0 | b | LinJ.03.0010 | Hypothetical protein, conserved |
|  |  |  |  |  |  |  | LinJ.03.0020 | Hypothetical protein, conserved |
|  |  |  |  |  |  |  | LinJ.03.0030 | D3-phosphoglycerate dehydrogenase, putative |
| Lin276E12 | 2.52 | 1.3 ± 0.4 | 0.033 | 0 | 2e-161 | a | LinJ.36.0550 | Hypothetical protein, conserved |
|  |  |  |  |  |  |  | LinJ.36.0560 | Protein phosphatase 2C |
|  |  |  |  |  |  |  | LinJ.36.0570 | Small nuclear ribonucleoprotein, putative |
|  |  |  |  |  |  |  | LinJ.36.0580 | Hypothetical protein, conserved |
| Lin37B5 | -2.75 | -1.5 ± 0.1 | 0.002 | 0 | 0 | c | LinJ.05.0750 | Hypothetical protein, conserved |
| Lin56A3 | -2.58 | -1.4 ± 0.1 | 0.002 | 0 | 0 | c | LinJ.35.0690 | Hypothetical protein, conserved |
|  |  |  |  |  |  |  | LinJ.32.1190 | Hypothetical protein, conserved |
| Lin73B8 | -2.39 | -1.3 ± 0.2 | 0.010 | 0 | 0 | c | LinJ.36.4440 | Hypothetical protein, conserved |
| Lin90E12 | -2.57 | -1.4 ± 0.3 | 0.017 | 0 | 0 | c | LinJ.21.1350 | Hypothetical protein, conserved |
| Lin91F9 | -2.26 | -1.2 ± 0.4 | 0.038 | 0 | 0 | c | LinJ.31.1630 | Hypothetical protein, unknown function |
| Lin101F3 | -2.09 | -1.1 ± 0.2 | 0.013 | 0 | 0 | c | LinJ.30.1870 | Hypothetical protein, conserved |
|  |  |  |  |  |  |  | LinJ.30.1880 | Hypothetical protein, conserved |
| Lin103C9 | -2.74 | -1.5 ± 0.3 | 0.014 | 0 | 0 | c | LinJ.23.1900 | Hypothetical protein, conserved |
|  |  |  |  |  |  |  | LinJ.30.2070 | Hypothetical protein, conserved |
| Lin109E10 | -9.09 | -3.2 ± 0.2 | 0.002 | 0 | - | c | LinJ.12.0330 | Hypothetical protein, unknown function |
| Lin118B11 | -12.1 | -3.6 ± 1.0 | 0.027 | 3e-123 | - | c | LinJ.32.0910 | Hypothetical protein, conserved |
| Lin125A7 | -2.27 | -1.2 ± 0.1 | 0.002 | 0 | 0 | c | LinJ.31.1360 | Hypothetical protein, conserved |
| Lin147A9 | -2.28 | -1.2 ± 0.3 | 0.009 | 7e-167 | 0 | c | LinJ.14.1590 | Sinaptojanin (N-terminal domain)/inositol 5’-phosphatase, putative |
| Lin153A11 | -2.17 | -1.1 ± 0.4 | 0.044 | - | 9e-114 | c | LinJ.30.3390 | 60S ribosomal protein L9 |
| Lin201D1 | -7.55 | -2.9 ± 0.8 | 0.025 | 2e-158 | - | c | LinJ.36.2970 | ATP-dependent DEAD/H helicase |
| Lin202D10 | -5.42 | -2.4 ± 0.8 | 0.034 | 0 | - | c | LinJ.31.2330 | Aminopeptidase metallopeptidase, Clan MA(E), familia M1 |
| Lin208D6 | -4.33 | -2.1 ± 0.3 | 0.005 | 0 | 0 | c | LinJ.29.1150 | Hypothetical protein, conserved |
