# Supplementary material for: Influence of the Microenvironment in the Transcriptome of Leishmania infantum Promastigotes: Sand Fly versus Culture
Source: PLoS Negl Trop Dis. 2016 May 10;10(5):e0004693. doi: 10.1371/journal.pntd.0004693 (PMC4862625; doi:10.1371/journal.pntd.0004693)
Supplement: S1 Text — (DOC) [file pntd.0004693.s010.doc]

**S1 Text. Detailed discussion of the differential gene expression profiles between Pro-Pper and Pro-Stat.**

The differences found in relative expression between Pro-Pper and Pro-Stat are discussed in this addenda and have been illustrated in Figure 4.

*Chromosome and regulation of gene expression at the post-transcriptional and post-translational levels.*

Relative transcript abundance of two different mismatch repair protein (MSH8 and MSH) genes between Pro-Pper and Pro-Stat is opposite and the A/G-specific adenine glycosylase gene (MYH), involved in base excision repair, is up-regulated in Pro-Pper. Future characterization of these repair proteins may lead to understand the relationship between their roles in promastigotes and the extracellular environment. The subunit 2 of the replication factor c (RFC2) is up-regulated in Pro-Pper at the transcript level. This gene is over-expressed at the beginning of the differentiation process of promastigotes to amastigotes *L. donovani* .

A histone H4 gene is also up-regulated in Pro-Pper. According to previous gene expression profiling, this gene is down-regulated in Pro-Stat with respect to logarithmic phase promastigotes (Pro-Log) in *L. infantum*  and up-regulated in Pro-Pper with respect to intracellular amastigotes . The histone deacetylase (HDAC) is also up-regulated in Pro-Pper. The up-regulation of a DNA-directed RNA polymerase-like protein (RNA Pol) and a DNA-directed RNA polymerase I subunit (RBP10) may be linked to this hypothetical change in histone acetylation. Although RBP10 is part of the RNA polymerase I complex, it is predicted to be shared by RNA polymerases I, II and III . It was described that histone acetylation marks origins of polycistronic transcription in *L. major* . Certain post-transcriptional regulation events at the trans-splicing level may also take place increasingly in Pro-Pper with respect to Pro-Stat, provided the up-regulation of the U2 snRNP and the pre-mRNA branch site p14 protein. The poly(A)-binding protein 3 (PABP3) gene is also up-regulated in Pro-Pper with respect to Pro-Stat, as well as in the metacyclic non-agglutinating subpopulation of Pro-Stat as reported . Consequently, higher PABP3 levels are probably linked to metacyclic promastigotes, where it may drive certain changes in RNA stability. Also, the up-regulation of the Lsm5p gene in Pro-Pper may participate in these hypothetical post-transcriptional regulation processes, as it has been described to be involved in RNA degradation and processing . The mitochondrial protein MP99 protein is up-regulated in Pro-Stat and participates in the RNA editing process characteristic of trypanosomatids .

Differences in regulation of translation may also exist between Pro-Pper and Pro-Stat. On the one hand, the eukaryotic translation initiation factor 4E (eIF4E), the elongation factor 1β (eEF1β), the 40S ribosomal protein S33, the 60S acidic ribosomal protein, the 60S ribosomal protein L23a and the histidine-tRNA synthetase are up-regulated in Pro-Pper. On the other hand, the ribosomal proteins SA, S2, S3a, S6, S29, L7, L12, L15, L21 and L44 are up-regulated in Pro-Stat, as well as the nuclear pore complex protein NUP155.

The following level of gene expression regulation is also affected probably, according to the up-regulation in Pro-Pper of protein folding and modification genes. These genes code for the translocation complex factors sec13 and sec14 (cytosolic), the subunit 1η from the T complex (Tcp1η), the cyclophilin 11 (CYP11), UDP-N-acetylglucosamine:phosphatidylinositol α-1,6 N-acetylglucosamine transferase (PIGA), an oligosaccharyl transferase (OSTL) acting on aspartic acid residues, the farnesyltransferase (FNTA) and the prenyl-protein specific carboxymethyltransferase acting on isoprenylcysteine residues (ICMT). The FNTA gene is over-expressed in Pro-Log with respect to Pro-Stat in *L. infantum*, whereas de ICMT is down-regulated in Pro-Stat with respect to intracellular amastigotes . PIGA is involved in the biosynthesis of the glycosylphosphatidylinositol anchor (GPI) for certain surface glycoproteins, proteophosphoglycans and glycolipids (see the subsection *Surface molecules* below).

Regarding proteolysis, the gene coding for a Zn-carboxypeptidase from the family M14 is up-regulated in Pro-Pper, as well as in metacyclic promastigotes from axenic culture . This protein may be extracellular, what is supported by TMHMM predictions (Supporting information 5). A signal aspartic peptidase gene from clan AD and family A22B is also up-regulated in Pro-Pper. This protein is a signal peptidase (PF04258) integral to membrane (GO:0016021) targeted to the presenilin signal peptide (IPR006639). The relative expression level of two different calpain-like cysteine peptidase genes is opposite between Pro-Pper and Pro-Stat. These genes are also different to the calpain-like protein over-expressed in metacyclic within the Pro-Stat population . These proteins are related to cell differentiation, cytoskeleton remodelling and signal transduction . The ubiquitin-proteasome protein degradation pathway may be more active also in Pro-Per than in Pro-Stat according to the up-regulation in Pro-Pper of the genes encoding the ubiquitin fusion-degradation protein (Ubq), the ubqUbqA-E1), two ubiquitin conjugating enzyme-like proteins (UbqC) and the E3 activating protein cullin. Conversely, the transcript levels of other ubiquitin conjugating enzyme (UbqC-E2) are higher in Pro-Stat. The relative expression level of the UbqC LinJ.33.2910 is also higher in Pro-Per with respect to intracellular amastigotes .

*Metabolism*

The glucosamine-6-phosphate N-acetyltransferase (GNAT), is up-regulated in Pro-Pper. This gene is also up-regulated in Pro-Log with respect to Pro-Stat in *L. infantum*  and in Pro-Stat with respect to both axenic amastigotes obtained by temperature increase plus pH decrease and intracellular amastigotes obtained by *in vitro* infection of the U937 cell line . It has been described that *L. major* amastigotes utilize hexosamine phosphates as a major carbon source and that these derivatives accumulate in parasites defective for the glucosamine-6-phospate deaminase. As hexosamines are probably produced by the macrophage and are toxic for the parasite, catabolism of these molecules is essential . As the GNAT catalyzes the reversible transfer of acetyl-CoA to GlcN-6-P yielding GlcNAc-6P, this enzyme may intervene in this catabolic process or be involved in the accumulation of GlcN derivatives in *L. infantum* promastigotes both in culture and in Pro-Pper. In fact, we have also described recently that the GNAT gene is up-regulated in Pro-Pper with respect to intracellular amastigotes .

In principle, the up-regulation in Pro-Stat of the invertase would somehow suggest a higher degree of sucrose utilization as a source of energy in promastigotes under culture conditions at the stationary phase. The same may be hypothesized for glucose, given that the glucose transporter 2 (GT2) is up-regulated in Pro-Stat also. However, gluconeogenesis may be also more active in those conditions, regarding the similar relative expression profile of the glycosomal phosphoenolpyruvate carboxykinase (gPEPCK). The UDP-glucose 4’ epimerase (Glc4’ep) is also over-expressed in Pro-Stat, what may be indicative of a higher rate of transformation of Glc into Gal in cultured forms than in the sand fly foregut. These data taken altogether suggest the utilization of hexoses as building blocks for glycoproteins and phosphoglycans or the pentose-phosphate pathway rather than as carbon and energy sources in Pro-Stat. Particularly, the Gal residues may be used for the biosynthesis of the LPG among other molecules. The exposed Gal residues of the LPG are blocked by arabinose residues in metacyclic promastigotes of *L. infantum* and *L. major* . Indeed, the fucose kinase (FK) gene, involved in arabinose phosphate biosynthesis , is up-regulated in Pro-Pper. GDP-arabinose-1-phosphate is the substrate for arabinosyltransferases to transfer arabinose residues to the LPG.

The p-nitrophenylphosphatase gene (PNPP) is up-regulated in Pro-Pper, and it was also reported to be up-regulated in axenic amastigotes with respect to Pro-Stat . There is evidence to support that the glyoxylate cycle and enzymes related to this pathway are present in *Leishmania* spp. In fact, it was already described in 1977 and it is also supported by results of annotation in the genome sequences . For example, the gene LinJ.03.0040 contains the alanine-glyoxylate transaminase activity (EC 2.6.1.44) (AGXT). Another example is the PNPP, which bears the EC 3.1.3.18 (phosphoglycolate phosphatase) activity. This activity is essential for photorespiration in plants and for the glycolate salvage pathway in humans, where it is found in many tissues . For example in red blood cells, where phosphoglycolate is an important activator of 2,3-bisphosphoglycerate hydrolysis, a major modifier of oxygen affinity of hemoglobin . The glyoxylate cycle may participate in glucolysis and gluconeogenesis, and also in glycine biosynthesis in *Leishmania* spp. .

The down-regulation in Pro-Pper of amino acid permease (AAP), α-ketoisovalerate dehydrogenase LinJ.23.0620 (α-KIVDH), δ−1-pirroline-5-carboxylate dehydrogenase (P5CDH), pirroline-5-carboxylate reductase (PC5R) and glutamate dehydrogenase (GluDH) suggests that proline, leucine, isoleucine and valine degradation rates are higher in Pro-Stat than in Pro-Pper. In addition, indoleacetate generation seems to be less active in Pro-Pper given the down-regulation of the α-ketoacid decarboxylase gene (IPDC) bearing the pyruvate (EC 4.1.1.1.) and indolepyruvate decarboxylase (EC.4.1.1.74.) activities. The nitrilase (EC 3.5.5.1) is also involved in tryptophan metabolism and is up-regulated in Pro-Stat. Most of the tryptophan degradation enzymes are not present in *Leishmania* spp. (KEGG database), but the IPDC gene is annotated. In fact, most of the aromatic amino acid oxidation pathways are missing and intermediate products are excreted . The same behavior is expected regarding lysine synthesis, as the succinyl-diaminopimelate desuccinylase (DAPDS) is down-regulated. Glutathione biosynthesis may be higher under stationary phase culture conditions as well, as γ-glutamylcysteine synthase (GSH1) is up-regulated in Pro-Stat. Taken as a whole; these data suggest that the major carbon and energy sources of Pro-Stat are amino acids.

A different scenario is observed in Pro-Pper, where genes related to lipid metabolic processes are up-regulated. One of the genes up-regulated in Pro-Pper is the dihydroxyacetone kinase 1-like protein gene (DHAK), which was found to be up-regulated during the differentiation process to amastigote and is involved in glycerolipid metabolism. The same expression profile has been found for the triacylglycerol lipase-like protein LinJ.31.0860 (TGL), the mitochondrial precursor of acyl-CoA dehydrogenase LinJ.07.0150 (mACDH) and the α-subunit of the mitochondrial trifunctional enzyme, suggesting an increase in β−oxidation of fatty acids. The gene encoding the 3-hydroxyacyl-ACP dehydratase (EHHADH) protein is up-regulated also in Pro-Pper and is involved in fatty acid biosynthesis. On the contrary, the lysophospholipase gene is up-regulated in Pro-Stat. The genes encoding the cytochrome c oxidase V (coxV) and the F1 α-subunit protein of the ATP synthetase complex are up-regulated in Pro-Pper. The mevalonate kinase gene (mvaK), involved in the biosynthesis of the isoprenoid backbone, is up-regulated in Pro-Pper, and ceramide biosynthesis may be more activated in Pro-Pper due to the up-regulation of the sphingolipid δ4-desaturase (DECS). An NAD(P)-dependent steroid dehydrogenase-like protein gene (NSDHL) is up-regulated in Pro-Stat instead. According to the enzymatic activity associated to this gene (EC 1.1.1.170), it participates in the biosynthesis of ergosterol and vitamin D3 in these parasites.

Certain genes involved in metablic processes of unknown exact function are up-regulated in Pro-Pper: three short chain dehydrogenase genes, an oxidoreductase-like protein gene and a mitochondrial carrier protein gene. A putative NUDIX hydrolase-like protein gene constitutively expressed in differentiation of Pro-Stat to axenic amastigotes in *L.major*  is up-regulated in Pro-Pper, what is consistent with the observations in metacyclic promastigotes in culture with respect to procyclics .

*Redox homeostasis.*

As a difference with *Leishmania* spp. cultured promastigotes, the motile stage choanomastigote of the monogenetic trypanosomatid *Crithidia fasciculata* over-expresses the trypanothione peroxidase (TryP) presumably due to a differentiation process in culture . TryP is not differentially regulated in *L. infantum* promastigotes within the sand fly foregut with respect to the culture conditions as confirmed by qRT-PCR (Table 2) and was not detected as differentially regulated between Pro-Log and Pro-Stat either .

*Transport.*

Two ATP synthetase genes are up-regulated between Pro-Pper and Pro-Stat: the above mentioned F1γ subunit and the subunit c of the vacuolar ATP synthetase (vATPSc). Regarding ABC transporters, a subfamily E member (ABCE1) is up-regulated in Pro-Pper, whereas the ABCC6 is up-regulated in Pro-Stat. Differential regulation of genes involved in transport of different inorganic and organic species has been found also. The Fe/Zn transporter (Fe/ZnT), the nucleoside transporter 1 (NsT1) and the pteridine transporter LinJ.06.1320 (PT) are up-regulated in Pro-Pper, whereas the GT2 and the AAP are up-regulated in Pro-Stat at the transcript level. The sodium stibogluconate resistance protein (SbGRP), the calcium motive P-type ATPase (Ca^2+^-ATPase) and a transporter-like protein gene are also up-regulated in Pro-Stat. Both the SbGRP and the GT2 were found to be up-regulated in Pro-Stat with respect to Pro-Log and the Ca^2+^-ATPase and the vesicle associated membrane protein (vamp) in intracellular amastigotes with respect to Pro-Stat in *L. infantum* at the transcript level . As already seen above, NUP155 and a transportin 2-like protein gene (TNP2), both involved in nucleocytoplasmic transport, are up-regulated in Pro-Stat.

With regard to vesicle-mediated transport, the following genes encoding coating and vesicle dynamics molecules are up-regulated in Pro-Pper: QA-SNARE, vamp (also up-regulated in Pro-Log with respect to Pro-Stat) , adaptor complex protein 3 δ subunit 1 (AP3δ1), clathrin coat assembly protein-like protein and vacuolar protein sorting-like protein (VPSL, involved in exocytosis). The opposite relative expression profile has been found for the COP-coated vesicle membrane protein erv25 gene. Overall, these findings suggest that vessicle trafficking may be increased in Pro-Pper.

*Intracellular signaling.*

Although the kinome of trypanosomatids has been well characterized , most of the signal transduction pathways have not been elucidated in these organisms yet . The following *L. infantum* genes involved in signaling are differentially regulated: the protein kinase (PK) LinJ.07.0410, the catalytic subunit A2 of the protein phosphatase 2B (PP2B-A2), a serine/threonine phosphatase type 5 (PP5), another serine/threonine PP, a protein kinase (PK), a mitogen-activated protein kinase (MAPK), the calmodulin, a calmodulin-like protein, the phosphatidylinositol 4-kinase (PI4K) and a β-propeller protein (β-prop) are up-regulated in Pro-Pper; the serine/threonine protein phosphatase 1 LinJ.34.0820/30 (PP1), two different PKs and a POLO-like kinase are up-regulated in Pro-Stat. The elucidation of signal transduction pathways is one of the major tasks pending for the explanation of environmental stimuli coming from the different hosts and the parasite response and survival. Also these parasites are adapted to axenic culture conditions and genes differentially expressed between Pro-Pper and Pro-Stat may contribute to explain how adaptation to these distinct environments takes place.

*Cytoskeleton.*

Organization of the actin cytoskeleton may be affected by the up-regulation in Pro-Pper of the actin (ACT) and the acting interacting proteins coronin (CRN12) and profilin (PFN) at the transcript level. Another actin-interacting protein gene (AIP) is up-regulated in Pro-Stat. The CRN12 is not only an actin-interacting protein , but also a kinesin-interacting protein in *Leishmania* spp., interacting with kinesin K39 during cytokinesis . However, the caltractin gene, probably involved in proper duplication and segregation of the centrosome, is up-regulated in Pro-Stat. In fact, it has been described that the disruption of this gene leads to impairment of basal body replication and abnormal cell cycle progression in *Leishmania* spp. . Moreover, regarding to the microtubule cytoskeleton and the flagellum, an α-tubulin gene is up-regulated in Pro-Stat, whereas one of the numerous kinesin genes and the dynein light chain lc6 flagellar outer arm gene, also up-regulated in Pro-Log with respect to Pro-Stat , and a tubulin-tyrosine ligase are up-regulated in Pro-Pper. Tyrosylation is one of the post-translational modifications of tubulins that lead to changes in microtubule organization, depending on the organisms. In the case of *Leishmania* spp., certain changes in the spatial distribution of tubulins triggered by acetylation and tyrosylation in the microtubule cytoskeleton may be linked to resistance to certain drugs . This resistance may be increased In Pro-Pper, the tubulin-tyrosine ligase gene is up-regulated.

*Biosynthesis of surface molecules*.

PIGA is involved in the biosynthesis of the GPI, the anchor of certain glycoproteins, the LPG, the membrane bound proteophosphoglycan (mPPG) and glycosylinositol phospholipids (GIPLs). GIPLs are the major surface molecules in amastigotes and serve as receptors for the host cell and as a shield for resistance against lysosomal hydrolases . The PI4K is involved in the biosynthesis of the PIGA reaction substrate phosphatidylinositol 1-phosphate. PIGA and PI4K are up-regulated in Pro-Pper, as well as a glycosyl transferase-like protein gene (GTL), an oligosaccharyl transferase-like protein (OSTL) and the dolichyl-P-Man:GDP-Man5GlcNAc2-PP-dolichyl α-1,3-mannosyltransferase (ALG3). ALG3 and OSTL are involved in N-glycan biosynthesis.

The hydrophilic surface protein (HASPA1) and the small hydrophilic endoplasmic reticulum protein (SHERP) genes are up-regulated in Pro-Stat. Although these molecules are important for development in the sand fly (Pro-Pper), according to this analysis, their relative expression at the transcript level is higher in culture (Pro-Stat). These genes are also over-expressed in Pro-Stat vs. Pro-Log . As Pro-Pper metacyclic promastigotes were obtained from the anterior part of *P. perniciosus* gut at the stomodeal valve (see Methods), this suggests that promastigotes increase the expression levels of HASP and SHERP molecules in culture due to the lack of certain environmental signals proper of the sand fly gut environment.

Regarding to the amastin superfamily, genes LinJ.08.0680/0690/0700/0710 and LinJ.34.2660 are up-regulated in Pro-Stat vs. Pro-Pper (this work) and vs. Pro-Log . Additionally, LinJ.08.0790/1320 and LinJ.24.1280 are up-regulated in Pro-Stat vs. Pro-Pper. Some of these genes are up-regulated when temperature is raised and pH decreased, both in axenic and intracellular amastigotes . These molecules may be over-expressed in advance to the differentiation process of promastigotes to amastigotes, what is in agreement with the pre-adaptation hypothesis . As the expression levels of these genes are higher in Pro-Stat than in Pro-Pper, the same as for HASP and SHERP is suggested: Pro-Stat are depleted of nutrients but sensing of the sand fly stomodeal valve promastigotes does not take place and somehow, they have to prepare in advance for infection in these conditions as Pro-Pper do.

**REFERENCES**

1. Saxena A, Lahav T, Holland N, Aggarwal G, Anupama A, et al. (2007) Analysis of the Leishmania donovani transcriptome reveals an ordered progression of transient and permanent changes in gene expression during differentiation. Mol Biochem Parasitol 152: 53-65.

2. Alcolea PJ, Alonso A, Gomez MJ, Moreno I, Dominguez M, et al. (2010) Transcriptomics throughout the life cycle of Leishmania infantum: high down-regulation rate in the amastigote stage. Int J Parasitol 40: 1497-1516.

3. Alcolea PJ, Alonso A, Gomez MJ, Postigo M, Molina R, et al. (2014) Stage-specific differential gene expression in Leishmania infantum: from the foregut of Phlebotomus perniciosus to the human phagocyte. BMC Genomics.

4. Nguyen TN, Schimanski B, Zahn A, Klumpp B, Gunzl A (2006) Purification of an eight subunit RNA polymerase I complex in Trypanosoma brucei. Mol Biochem Parasitol 149: 27-37.

5. Thomas S, Green A, Sturm NR, Campbell DA, Myler PJ (2009) Histone acetylations mark origins of polycistronic transcription in Leishmania major. BMC Genomics 10: 152.

6. Alcolea PJ, Alonso A, Sanchez-Gorostiaga A, Moreno-Paz M, Gomez MJ, et al. (2009) Genome-wide analysis reveals increased levels of transcripts related with infectivity in peanut lectin non-agglutinated promastigotes of Leishmania infantum. Genomics 93: 551-564.

7. Yang Y, Meier UT (2003) Genetic interaction between a chaperone of small nucleolar ribonucleoprotein particles and cytosolic serine hydroxymethyltransferase. J Biol Chem 278: 23553-23560.

8. Panigrahi AK, Schnaufer A, Ernst NL, Wang B, Carmean N, et al. (2003) Identification of novel components of Trypanosoma brucei editosomes. RNA 9: 484-492.

9. Ono Y, Sorimachi H, Suzuki K (1998) Structure and physiology of calpain, an enigmatic protease. Biochem Biophys Res Commun 245: 289-294.

10. Sato K, Kawashima S (2001) Calpain function in the modulation of signal transduction molecules. Biol Chem 382: 743-751.

11. Alcolea PJ, Alonso A, Gomez MJ, Sanchez-Gorostiaga A, Moreno-Paz M, et al. (2010) Temperature increase prevails over acidification in gene expression modulation of amastigote differentiation in Leishmania infantum. BMC Genomics 11: 31.

12. Naderer T, Heng J, McConville MJ (2010) Evidence that intracellular stages of Leishmania major utilize amino sugars as a major carbon source. PLoS Pathog 6: e1001245.

13. Sacks DL, Hieny S, Sher A (1985) Identification of cell surface carbohydrate and antigenic changes between noninfective and infective developmental stages of Leishmania major promastigotes. J Immunol 135: 564-569.

14. Saxena A, Worthey EA, Yan S, Leland A, Stuart KD, et al. (2003) Evaluation of differential gene expression in Leishmania major Friedlin procyclics and metacyclics using DNA microarray analysis. Mol Biochem Parasitol 129: 103-114.

15. Novozhilova NM, Bovin NV (2009) D-Arabinose Methabolism: Characterization of Bifunctional Arabinokinase/Pyrophosphorylase of Leishmania major. Acta Naturae 1: 81-83.

16. Rochette A, Raymond F, Corbeil J, Ouellette M, Papadopoulou B (2009) Whole-genome comparative RNA expression profiling of axenic and intracellular amastigote forms of Leishmania infantum. Mol Biochem Parasitol 165: 32-47.

17. Lahav T, Sivam D, Volpin H, Ronen M, Tsigankov P, et al. (2011) Multiple levels of gene regulation mediate differentiation of the intracellular pathogen Leishmania. FASEB J 25: 515-525.

18. Simon MW, Martin E, Mukkada AJ (1978) Evidence for a functional glyoxylate cycle in the leishmaniae. J Bacteriol 135: 895-899.

19. Mukkada AJ (1977) Tricarboxylic acid and glyoxylate cycles in the Leishmaniae. Acta Trop 34: 167-175.

20. Ivens AC, Peacock CS, Worthey EA, Murphy L, Aggarwal G, et al. (2005) The genome of the kinetoplastid parasite, Leishmania major. Science 309: 436-442.

21. Peacock CS, Seeger K, Harris D, Murphy L, Ruiz JC, et al. (2007) Comparative genomic analysis of three Leishmania species that cause diverse human disease. Nat Genet 39: 839-847.

22. Beutler E, West C (1980) An improved assay and some properties of phosphoglycolate phosphatase. Anal Biochem 106: 163-168.

23. Barker RF, Hopkinson DA (1978) Genetic polymorphism of human phosphoglycolate phosphatase (PGP). Ann Hum Genet 42: 143-151.

24. Rose ZB, Liebowitz J (1970) 2,3-diphosphoglycerate phosphatase from human erythrocytes. General properties and activation by anions. J Biol Chem 245: 3232-3241.

25. Opperdoes FR, Michels PA (2008) The metabolic repertoire of Leishmania and implications for drug discovery. In: Myler P, Fassel N, editors. Leishmania after the genome. Norfolk: Caister Academic Press. pp. 123-158.

26. Rosenzweig D, Smith D, Opperdoes F, Stern S, Olafson RW, et al. (2008) Retooling Leishmania metabolism: from sand fly gut to human macrophage. FASEB J 22: 590-602.

27. Leifso K, Cohen-Freue G, Dogra N, Murray A, McMaster WR (2007) Genomic and proteomic expression analysis of Leishmania promastigote and amastigote life stages: the Leishmania genome is constitutively expressed. Mol Biochem Parasitol 152: 35-46.

28. Akopyants NS, Matlib RS, Bukanova EN, Smeds MR, Brownstein BH, et al. (2004) Expression profiling using random genomic DNA microarrays identifies differentially expressed genes associated with three major developmental stages of the protozoan parasite Leishmania major. Mol Biochem Parasitol 136: 71-86.

29. Alcolea PJ, Alonso A, García-Tabares F, Toraño A, Larraga V (2014) An insight into the proteome of Crithidia fasciculata choanomastigotes as a comparative approach to axenic growth, peanut lectin agglutination and differentiation of Leishmania spp. promastigotes. PLoS ONE Under review.

30. Parsons M, Worthey EA, Ward PN, Mottram JC (2005) Comparative analysis of the kinomes of three pathogenic trypanosomatids: Leishmania major, Trypanosoma brucei and Trypanosoma cruzi. BMC Genomics 6: 127.

31. Parsons M, Ruben L (2000) Pathways involved in environmental sensing in trypanosomatids. Parasitol Today 16: 56-62.

32. Nayak RC, Sahasrabuddhe AA, Bajpai VK, Gupta CM (2005) A novel homologue of coronin colocalizes with actin in filament-like structures in Leishmania. Mol Biochem Parasitol 143: 152-164.

33. Sahasrabuddhe AA, Nayak RC, Gupta CM (2009) Ancient Leishmania coronin (CRN12) is involved in microtubule remodeling during cytokinesis. J Cell Sci 122: 1691-1699.

34. Selvapandiyan A, Debrabant A, Duncan R, Muller J, Salotra P, et al. (2004) Centrin gene disruption impairs stage-specific basal body duplication and cell cycle progression in Leishmania. J Biol Chem 279: 25703-25710.

35. Chavan HD, Singh G, Dey CS (2007) Confocal microscopic investigation of tubulin distribution and effect of paclitaxel on posttranslationally modified tubulins in sodium arsenite resistant Leishmania donovani. Exp Parasitol 116: 320-326.

36. Blackwell JM, Ezekowitz RA, Roberts MB, Channon JY, Sim RB, et al. (1985) Macrophage complement and lectin-like receptors bind Leishmania in the absence of serum. J Exp Med 162: 324-331.

37. Depledge DP, Evans KJ, Ivens AC, Aziz N, Maroof A, et al. (2009) Comparative expression profiling of Leishmania: modulation in gene expression between species and in different host genetic backgrounds. PLoS Negl Trop Dis 3: e476.
